# Lysosome‐dependent FOXA1 ubiquitination contributes to luminal lineage of advanced prostate cancer

**DOI:** 10.1002/1878-0261.13497

**Published:** 2023-08-21

**Authors:** Sherly I. Celada, Guoliang Li, Lindsay J. Celada, Wenfu Lu, Thanigaivelan Kanagasabai, Weiran Feng, Zhen Cao, Nazifa Salsabeel, Ninghui Mao, LaKendria K. Brown, Zaniya A. Mark, Michael G. Izban, Billy R. Ballard, Xinchun Zhou, Samuel E. Adunyah, Robert J. Matusik, Xiaofei Wang, Zhenbang Chen

**Affiliations:** ^1^ Department of Biochemistry, Cancer Biology, Neuroscience and Pharmacology Meharry Medical College Nashville TN USA; ^2^ Department of Biological Sciences Tennessee State University Nashville TN USA; ^3^ Department of Medicine Baylor College of Medicine Houston TX USA; ^4^ Human Oncology and Pathogenesis Program Memorial Sloan Kettering Cancer Center New York NY USA; ^5^ Weill Cornell Graduate School of Medical Sciences Weill Cornell Medicine New York NY USA; ^6^ Department of Pathology, Anatomy and Cell Biology Meharry Medical College Nashville TN USA; ^7^ Department of Pathology University of Mississippi Medical Center Jackson MS USA; ^8^ Department of Urology Vanderbilt University Medical Center Nashville TN USA

**Keywords:** FOXA1, luminal lineage, prostate cancer, SKP2, ubiquitination

## Abstract

Changes in FOXA1 (forkhead box protein A1) protein levels are well associated with prostate cancer (PCa) progression. Unfortunately, direct targeting of FOXA1 in progressive PCa remains challenging due to variations in FOXA1 protein levels, increased *FOXA1* mutations at different stages of PCa, and elusive post‐translational FOXA1 regulating mechanisms. Here, we show that SKP2 (S‐phase kinase‐associated protein 2) catalyzes K6‐ and K29‐linked polyubiquitination of FOXA1 for lysosomal‐dependent degradation. Our data indicate increased SKP2:FOXA1 protein ratios in stage IV human PCa compared to stages I–III, together with a strong inverse correlation (*r* = −0.9659) between SKP2 and FOXA1 levels, suggesting that SKP2–FOXA1 protein interactions play a significant role in PCa progression. Prostate tumors of *Pten/Trp53* mice displayed increased Skp2–Foxa1–Pcna signaling and colocalization, whereas disruption of the Skp2–Foxa1 interplay in *Pten/Trp53/Skp2* triple‐null mice demonstrated decreased Pcna levels and increased expression of Foxa1 and luminal positive cells. Treatment of xenograft mice with the SKP2 inhibitor SZL P1‐41 decreased tumor proliferation, SKP2:FOXA1 ratios, and colocalization. Thus, our results highlight the significance of the SKP2–FOXA1 interplay on the luminal lineage in PCa and the potential of therapeutically targeting FOXA1 through SKP2 to improve PCa control.

## Graphical abstract

FOXA1 is a transcriptional activator for steroid hormone receptors that can induce luminal epithelial cell differentiation in the prostate. Targeting post‐translational regulatory mechanisms for FOXA1 through SKP2 inhibition (SZL P1‐41) reduced prostate tumor proliferation, and restored luminal phenotypes and FOXA1 levels, supporting enhanced therapeutic sensitivity in advanced stages of PCa.

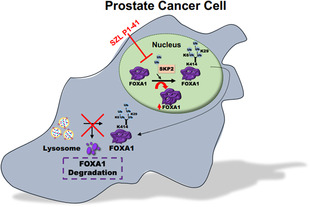

**Abbreviations**
ADT: androgen deprivation therapy
AP: anterior prostate
AR: androgen receptor
co‐IP: co‐immunoprecipitation
CRPC: castration‐resistant PCa
EV: empty vector
FKHD: forkhead domain
FOXA1: forkhead box protein A1
i.p.: intraperitoneal
IB: immunoblot
IF: immunofluorescence
KD: knockdown
KO: knockout
MEFs: mouse embryonic fibroblasts
MFI: mean fluorescence intensity
NE: neuroendocrine
OE: overexpression
PCa: prostate cancer
PCNA: proliferating cell nuclear antigen
SKP2: S‐phase kinase‐associated protein‐2
SYP: synaptophysin
TAD: transactivating domain
TMA: tissue microarray
t‐NEPC: treatment‐emergent neuroendocrine PCa
Ub: ubiquitin
WT: wild type

## 1 Introduction

Prostate cancer (PCa) is one of the most frequently diagnosed malignancies among American males, and one of the leading causes of cancer‐associated deaths in the USA [1, 2]. Androgen deprivation therapy (ADT) remains the primary method of treatment due to its efficacy in targeting the steroid hormone receptor androgen receptor (AR), an essential mediator of PCa development and progression. Unfortunately, resistance to androgen deprivation is frequently experienced by patients who progress to a more aggressive and lethal stage of PCa known as castration‐resistant PCa (CRPC) [3, 4]. Tumor growth of androgen‐independent PCa is also characterized by ADT desensitization and can undergo neuroendocrine (NE) differentiation leading to treatment‐emergent neuroendocrine PCa (t‐NEPC), a fatal and rapidly progressive malignancy [5]. Therefore, identifying facilitators of PCa progression is essential for developing effective treatments.

Forkhead box protein A1 (FOXA1) is a transcriptional activator for steroid hormone receptors such as AR [6]. As a regulator of prostate luminal epithelial cell differentiation, FOXA1 was shown to promote the transition from compact to permissive chromatin‐inducing gene expression [7, 8, 9]. In addition, frequent *FOXA1* mutations are associated with increased cellular proliferation leading to prostate tumorigenesis [10, 11]. In CRPC, nuclear localization and overexpression of FOXA1 enhanced tumor growth and metastases by prompting cell cycle progression [12, 13, 14], whereas decreased luminal FOXA1 is associated with the promotion of NEPC [15, 16]. However, it is still not well understood how these opposing effects result in PCa progression. These lines of evidence underscore the importance of investigating FOXA1 regulatory mechanisms in advanced PCa.

Overexpression of the S‐phase kinase‐associated protein‐2 (SKP2), the substrate recognition component of the SCF E3 ubiquitin ligase complex, is also associated with PCa recurrence and poor patient survival [17, 18]. SKP2 is a proto‐oncogene that inhibits cell cycle arrest and cellular senescence by regulating the tumor suppressor p27 [19]. Loss of SKP2 in CRPC was shown to repress prostate tumorigenesis and metastasis [20, 21, 22]. However, the role of SKP2 in NEPC is not well defined. Since both SKP2 and FOXA1 play an essential role in PCa progression, we hypothesized that the SKP2–FOXA1 interplay regulates PCa outcome. Our findings revealed a novel signaling pathway in advanced PCa by which SKP2 catalyzes the non‐canonical ubiquitination of FOXA1 to induce its lysosomal‐dependent degradation. Our results *in vivo* also indicated that greater Skp2–Foxa1–Pcna colocalization in prostate tumors of *Pten/Trp53* mice, an *in vivo* model for advanced PCa, promoted PCa progression, whereas loss of Skp2 in *Pten* ^*pc−/−*^; *Trp53* ^*pc−/−*^; *Skp2* ^*−/−*^ reduced tumor burden by stabilizing Foxa1 protein and luminal phenotypes while simultaneously decreasing Pcna levels. Further, pharmaceutical inhibition of SKP2 E3 ligase activity using SZL P1‐41 in C4‐2B and 22Rv1 nude mice impaired SKP2–FOXA1 interactions subsequently reducing tumor proliferation. Thus, our data implicate the SKP2–FOXA1 protein interplay as a valuable predictor of patient prognosis and its targeting a promising strategy against advanced stages of PCa.

## 2 Materials and methods

### 2.1 Cell line authentication

All human PCa cell lines were obtained from the American Type Culture Collection, ATCC (www.atcc.org; Manassas, VA, USA) including 22Rv1 (CRL‐2505; RRID: CVCL_1045) and C4‐2B (CRL‐3315; RRID: CVCL_4784). The human embryonic kidney 293T (CRL‐11268; RRID: CVCL_0063) cell line was obtained from ATCC. Mouse embryonic fibroblasts (MEFs) were prepared according to the methods previously described [23]. All cell lines were subjected to a complete validation process and were authenticated by performing Short Tandem Repeat (STR) profiling analysis at least once per year. Experiments and analysis performed using all cell lines were completed in triplicate.

### 2.2 Cell culture

Human PCa cell lines including 22Rv1 (CRL‐2505; RRID: CVCL_1045) and C4‐2B (CRL‐3315; RRID: CVCL_4784) were cultured in RPMI 1640 (Gibco, Billings, MT, USA) medium supplemented with 10% FBS (Gibco) and 1% Pen/Strep (Gibco) in an incubator with 5% CO_2_ at 37 °C. Human embryonic kidney 293T (CRL‐11268; RRID: CVCL_0063) cell lines and mouse embryonic fibroblasts (MEFs) were cultured in DMEM (Gibco) medium supplemented with 10% FBS (Gibco) and 1% Pen/Strep (Gibco) at 37 °C with 5% CO_2_. All cell lines were subjected to tracked passages during the cell culture process and all cell line cultures were regularly screened for mycoplasma contamination using a PCR Mycoplasma detection kit (MD Bioproducts, Oakdale, MN, USA).

### 2.3 SKP2 overexpression and knockdown

Changes in substrate gene expression were determined 48–72 h after transfection using immunoblot as described [24]. For SKP2 overexpression, pcDNA3‐Myc‐SKP2 plasmid was leveled with pcDNA3.1 empty vector in all samples of C4‐2B, 22Rv1, and 293T cell lines. To develop stable SKP2 KD C4‐2B and 22Rv1 cells, lentiviral particles carrying shSKP2 #1, shSKP2 #2 or scrambled plasmid sequences were generated using 293FT cells. Briefly, 293FT cells were co‐transfected with a three‐plasmid system including 5 μg of shSKP2 RNA or scrambled plasmids, 3 μg of psPAX2 packaging plasmid, and 2 μg of pMD2.G envelope plasmid with Lipofectamine 2000 (Thermo Fisher Scientific, Waltham, MA, USA). The viral supernatant was gathered and filtered using a 0.45‐μm filter approximately 48 h after transfection. After additional 48 h, infected cells were selected with puromycin (2 μg·mL^−1^) for 7 days.

### 2.4 Xenograft assays in nude mice

All procedures involving *in vivo* xenograft mice were conducted in accordance with the guidelines set forth by the Institutional Animal Care and Use Committee (IACUC) and IACUC‐approved study and protocols at Meharry Medical College (A3420‐01) in compliance with all relevant ethical regulations. All xenograft mice were housed under pathogen‐free conditions within the animal care facility at Meharry Medical College. All mice had free access to food and water. Male nude mice (Charles River Laboratory, Wilmington, MA, USA) at 8 weeks of age were subcutaneously injected with C4‐2B or 22Rv1 (2 × 10^6^) human PCa cells suspended in 200 μL of PBS with 50% Matrigel (BD Biosciences, Franklin Lakes, NJ, USA) in the dorsal flank of the mice. Mice were randomly divided into groups (*n* = 5 per group) and treated with 200 μL of a vehicle (DMSO control) or SZL P1‐41 (30 mg·kg^−1^; three times per week; intraperitoneal, i.p. injection) for 30 days. Tumor growth was measured using a digital caliper three times a week and treatment was administered upon tumor volume reaching ~ 100 mm^3^. Proceeding treatment, mice were euthanized, xenograft tumors were excised and tumor sections were fixed with formalin and embedded in paraffin for IHC and fluorescence staining.

### 2.5 Mutant mice and analysis

*Pten* ^*loxP/LoxP*^; *Trp53* ^*loxP/loxP*^; *Skp2* ^*−/−*^ and *Probasin‐Cre4* mutant mice were generated as previously described [25]. Briefly, female mice containing *LoxPten*, *LoxTrp53*, and *Skp2* ^*+/−*^ were crossed with males containing the alleles for *LoxPten*, *LoxTrp53*, *Skp2* ^*+/−*^, and *Probasin‐Cre4* in order to produce male *Pten* ^*loxP/loxP*^; *Trp53* ^*loxP/loxP*^; *Probasin‐Cre4 (Pten* ^*pc−*/−^; *Trp53* ^*pc*−/−^) conditional double KO mice or male *Pten* ^*loxP/loxP*^; *Trp53* ^*loxP/loxP*^; *Skp2* ^*−/−*^; *Probasin‐Cre4* (*Pten* ^*pc−*/−^; *Trp53* ^*pc−/−*^; *Skp2* ^*−/−*^) conditional triple KO mice. Mouse genotypes were verified through polymerase chain reaction (PCR) by mouse DNA and primers (Table S1). The following PCR program was used: 95 °C for 30 s, 57 °C for 1 min, 72 °C for 1 min for 30 cycles, and 72 °C for 5 min using BioRad thermal cycler. All animal handling and experiments were performed in accordance with Institutional Animal Care and Use Committee (IACUC)‐approved study and protocols at Meharry Medical College (A3420‐01). All experimental animals were maintained in a mixed genetic background of C57BL/6J × 129/Sv × FVB and housed within the animal care facility at Meharry Medical College with free access to food and water. Prostate tissues were collected and fixed in 10% neutral‐buffered formalin (Sigma‐Aldrich, St. Louis, MO, USA) overnight, washed, and preserved in 70% ethanol at 4 °C before exposure to ethanol dehydration and paraffin embedding (HistoWiz Inc, Long Island Ciy, NY, USA).

### 2.6 Prostate organoid

Murine prostate organoids were derived as previously described [26]. Briefly, murine prostates were harvested from *Probasin‐Cre4; Pten* ^*loxP/loxP*^; *Trp53* ^*loxP/loxP*^ genotype mice. Prostates were then digested with Collagenase Type II (Gibco) at 37 °C for 2 h followed by exposure to TrypLE (Gibco) at 37 °C for single‐cell suspension. Y‐27632 (10 μm) was used as a supplement to inhibit anoikis. Epithelial cells isolated from the prostate (35 μL drops) were embedded in basement membrane extracts (Matrigel, Corning, NY, USA) and organoid medium including advanced DMEM/F12 (Thermo Fisher Scientific) supplemented with B27 (Gibco), 10 mm Hepes (Gibco), 10 μm GlutaMAX (Gibco), 1.25 mm N‐Acteyl Cysteine (Millipore Sigma, Burlington, MA, USA), Pen/Strep (Gibco), 50 ng·mL^−1^ epithelial growth factor, 5% (v/v) R‐spondin 1, 10% (v/v) Noggin, 1 nm DHT (Millipore Sigma), 200 nm A83‐001 (Tocris Bioscience, Bristol, UK). Prostate organoids were trypsinized using TRYPLE (Gibco) and passaged weekly.

### 2.7 Real‐time quantitative PCR

Real‐time quantitative PCR was performed by first extracting total RNA using an RNeasy Mini Kit (Qiagen, Hilden, Germany) from the cells of interest. A total of 5 μg of RNA was used for cDNA synthesis using a reverse transcription SuperScript III first‐strand synthesis kit (Invitrogen, Waltham, MA, USA). A Bio‐Rad CFX96 real‐time system was used to perform qRT‐PCR in triplicate as previously described [27]. Forward and reverse primer sequences used are listed in Table S2.

### 2.8 Immunofluorescence (IF) and immunohistochemistry (IHC)

For IF staining, C4‐2B and 22Rv1 cells were grown on coverslips and stimulated with 10 nm DHT for 30 min and then fixed with methanol at −20 °C. Cells were then incubated with primary antibodies including mouse anti‐SKP2 (2 μg·mL^−1^, Invitrogen, 32‐3300), rabbit anti‐FOXA1 (1 : 250, Abcam, Cambridge, UK, ab170933), rat anti‐LAMP2 (1 : 1000, Invitrogen, MA1‐165), and mouse anti‐Myc tag (5 μg·mL^−1^, Abcam, ab32) as previously described [24]. IHC staining for anterior prostate (AP) mouse tissue sections and for human PCa tissue microarray (TMA) were performed as previously described [28]. Briefly, paraffin‐embedded sections (5 μm thickness) were de‐paraffinized in xylene 3× (10 min each) and rehydrated in graded alcohol and boiled in citrate buffer, pH 6.0 for 15 min for antigen retrieval. Samples were quenched in 3% H_2_O_2_ and placed in blocking buffer (10% FBS and 1× PBS containing 0.1% Triton X‐100 and 1% BSA) for 1 h. The sections were then exposed to the primary antibodies overnight at 4 °C: rabbit anti‐SKP2 (1 : 50, Abcam, ab183039) and rabbit anti‐FOXA1 (1 : 1000, Abcam, ab170933). Next, the samples were stained with biotinylated secondary antibodies (1 h) and visualized using an ABC kit and chromogen DAB substrate (Vector Labs). Nuclei were counterstained with Gill 3 Hematoxylin (Thermo Fisher Scientific). Human prostate tissue microarray slides were purchased from Biomax (35 PCa patients and 5 normal cases) and TriStar Technology Group (45 hormone refractory PCa patients and 5 normal cases). The scores for FOXA1 and SKP2 staining were graded as follows: 0 (negative), 1 (weak), 2 (moderate), and 3 (strong) according to the intensity of staining [29].

### 2.9 Flow cytometry

Proceeding sample collection, cells were fixed and permeabilized using Intracellular Fixation and Permeabilization Buffer Set (eBiosciences) for 1 h at 4 °C as previously described [30]. Briefly, samples were washed and stained with anti‐ubiquitin (1 : 50, Invitrogen, LF‐MAO118) for 1 h at 4 °C. For secondary antibody conjugation, goat anti‐mouse IgG Pacific Orange (1 μg·mL^−1^, Invitrogen, P31585) was added. Following this step, samples were stained with anti‐FOXA1 (1 : 50, Abcam, ab1709323) and conjugated to donkey anti‐rabbit Alexa Fluor 488 (1/2000, Abcam, ab150073). All Samples were analyzed using BD LSRFortessa (BD Biosciences). Forward and side scatter properties were used for gating singlets and live cells. flowjo x software (Tree Star Inc, Ashland, OR, USA) was used to analyze data with a minimum of 150 000 events acquired per sample. MFI was acquired using calibrator beads for FACS machine calibration.

### 2.10 Western blot, half‐life, immunoprecipitation, and ubiquitination assay

Cell lysates were prepared using RIPA lysis buffer [1× PBS, 1% Nonidet P‐40/Triton X‐100, 0.5% sodium deoxycholate, 2 mm EDTA, 0.1% SDS and protease inhibitor cocktail (Roche, Basel, Switzerland)]. The following antibodies were used for immunoblot analysis: mouse anti‐SKP2 (2 μg·mL^−1^, Invitrogen, 32‐3300), rabbit anti‐FOXA1 (1 : 1000, Cell Signaling Technology, Danvers, MA, USA, EZE8W), mouse anti‐β‐actin (1 : 10 000, Sigma‐Aldrich, A5441), and rabbit anti‐V5‐Tag (1 : 1000, Cell Signaling, D3H8Q). Protein stability and half‐life were determined by exposing the cells of interest to 100 μg·mL^−1^ of cycloheximide (CHX, Sigma‐Aldrich) and collecting lysates during the indicated time points for immunoblot blot analysis. For immunoprecipitation (IP), post 24‐h transfection, cells were exposed to RIPA buffer [1× PBS, 1% Nonidet P‐40/Triton X‐100, 0.5% sodium deoxycholate, 2 mm EDTA, 0.1% SDS and protease inhibitor cocktail (Roche)], sonicated, and centrifuged. Protein A/G plus‐agarose beads (Santa Cruz Biotechnology, Dallas, TX, USA) were used for immunoprecipitation with primary antibodies including mouse anti‐c‐Myc (1 : 50, Santa Cruz, sc‐40), mouse anti‐Flag M2 (1 : 50, Sigma‐Aldrich, F3165), rabbit anti‐SKP2 (1 : 50, Cell Signaling, D3G5), and rabbit anti‐FOXA1 (1 : 50, Cell Signaling, EZE8W) or normal IgG (1 : 50, Cell Signaling, 2729) control at 4 °C for 16 h. Subsequently, the beads were washed in PBS, boiled, and subjected to western blotting. *In vivo* ubiquitination was performed by transfecting HEK293T, C4‐2B, and 22Rv1 cells with epitope‐tagged plasmids including pcDNA3‐Myc‐SKP2 (Addgene, Plasmid #19947), FOXA1 full‐length WT and truncated plasmids as previously generated [28], FOXA1 lysine (K) mutated plasmids (GenScript, Piscataway, NJ, USA), HA‐Ub (Addgene, Plasmid #18712), pRK5‐HA‐Ub‐K6 (Addgene, Plasmid #22900), pRK5‐HA‐Ub‐K6R (Addgene, Plasmid #121153), pRK5‐HA‐Ub‐K11 (Addgene, Plasmid #22901), pRK5‐HA‐Ub‐K27 (Addgene, Plasmid #22902), pRK5‐HA‐Ub‐K29 (Addgene, Plasmid #22903), pRK5‐HA‐Ub‐K29R (Addgene, Plasmid #17602), pRK5‐HA‐Ub‐K33 (Addgene, Plasmid #17607), pRK5‐HA‐Ub‐K48 (Addgene, Plasmid #17605), pRK5‐HA‐Ub‐K348R (Addgene, Plasmid #17604), pRK5‐HA‐Ub‐K63 (Addgene, Plasmid #17606).

### 2.11 Datasets and statistical analysis

Reference datasets were prepared from previously conducted studies on prostate cancer and were retrieved from the National Center for Biotechnology Information gene expression omnibus (GEO) with accession numbers GSE25136 as previously described [31] and from cBioPortal for cancer genomics (cbioportal.org) including the Shancheng et al. and Beltran et al. datasets. The respective normalized expression values were plotted using graphpad prism (San Diego, CA, USA). All experiments were performed independently at least three times. Values were expressed as mean ± SEM. Significance was analyzed using two‐tailed Student's *t‐*test with a value of *P* < 0.05 being considered as statistically significant.

## 3 Results

### 3.1 FOXA1 levels are inversely correlated with SKP2 in human prostate cancer

**Fig. 1:**
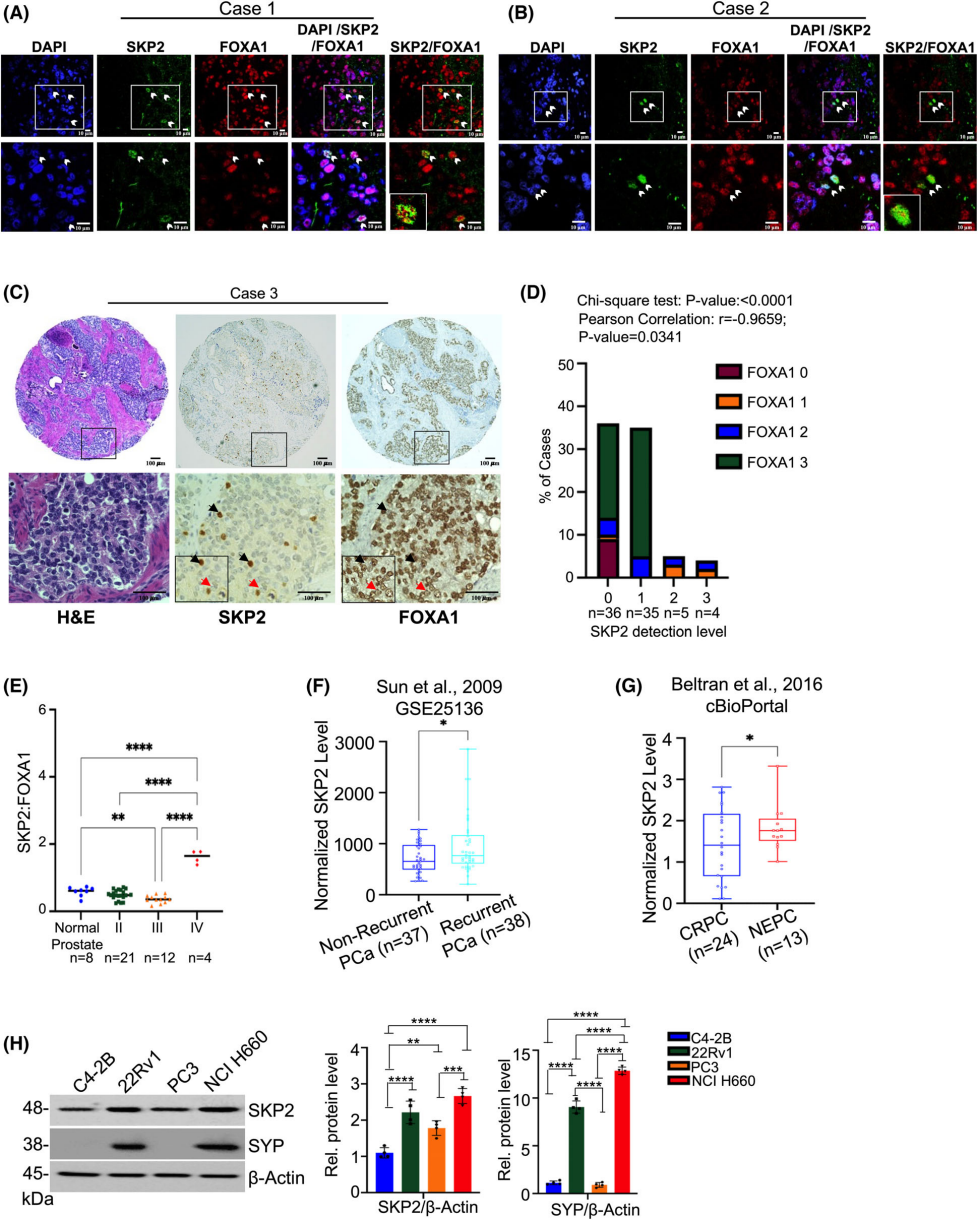
Enhanced SKP2:FOXA1 interaction correlates with advanced PCa in human specimens. (A, B) Immunofluorescence (IF) images demonstrate the colocalization of SKP2 (green) and FOXA1 (red) proteins in primary prostate tumors. White arrows represent SKP2 and FOXA1 protein colocalization. Scale bars are 10 μm. (C) Immunohistochemistry (IHC) staining for SKP2 and FOXA1 in tissue microarray (TMA) of primary prostate tumors (*n* = 80). The representative images shown are H&E, SKP2, and FOXA1. Black arrows represent high SKP2 and low FOXA1 protein levels. Red arrows represent low SKP2 and high FOXA1 protein. Scale bars are 100 μm. (D) Intensity scores for SKP2 and FOXA1 staining were graded as 0, 1, 2, and 3 and the respective statistical significance was determined by Chi‐square test (Fig. S2) and Pearson correlation coefficient. (E) SKP2‐to‐FOXA1 ratio (SKP2:FOXA1) for IHC staining for SKP2 and FOXA1 TMA of primary prostate tumors (*n* = 45). (F, G) SKP2 mRNA expression is elevated in recurrent PCa (GSE25136; *P* = 0.0459) and neuroendocrine PCa (NEPC) (*P* = 0.0224). CRPC refers to Castration‐resistant PCa. Previously published PCa gene expression datasets were retrieved from GEO database (ncbi.nlm.nih.gov/gds/) and cbioportal (cbioportal.org), respectively. The corresponding normalized SKP2 levels were plotted. (H) Immunoblot analysis for several PCa cell lines. Quantification analysis of the relative protein levels for SKP2 and SYP is displayed to the right (*n* = 4). Comparison between groups was performed using Student's *t*‐test. Bars indicate SEM. *P < 0.05, **P < 0.01, ***P < 0.001, ****P < 0.0001.

To evaluate if an association exists between SKP2 and FOXA1, we used tissue microarray (TMA) from normal and PCa specimens. Double IHC staining revealed simultaneous nuclear colocalization between SKP2 (*red*) and FOXA1 (*brown*) within normal (Fig. S1a) and PCa sections (Fig. S1b). This was confirmed by double‐fluorescent immunohistochemistry of TMA cores also identifying nuclear colocalization (*yellow*) between SKP2 (*green*) and FOXA1 (*red*) protein in patients with varying degrees of PCa (Fig. 1A,B). Additionally, we consistently observed an inverse correlation between SKP2 and FOXA1 levels in TMA tissue cores (Fig. 1C; *black* arrows indicate high SKP2 and low FOXA1 while *red* arrows indicate low SKP2 and high FOXA1). Pearson correlation analysis identified a strong negative correlation (Fig. 1D; Fig. S2, *r* = −0.9659, *P* = 0.0341) between SKP2 and FOXA1. Chi‐square analysis (Fig. 1D; Fig. S2, *P* ≤ 0.0001) for TMA staining intensity scores confirmed an inverse relation between SKP2 and FOXA1. About 37% of TMA cores displayed a FOXA1 intensity score of 3 that corresponded with SKP2 detection levels of 1, whereas 11% of TMA cores displayed SKP2 intensity scores between 2 and 3 which corresponded with FOXA1 detection levels between 0 and 2 (Fig. 1D; Fig. S2). Furthermore, significantly elevated SKP2:FOXA1 (SKP2 intensity/FOXA1 intensity) was detected in TMA cores at stage IV PCa compared to normal prostate tissue and stages II and III of PCa (Fig. 1E, mean = 1.6, *P* < 0.0001), suggesting that the SKP2‐FOXA1 interplay increases with PCa severity. Public datasets [32, 33, 34] also revealed a significant up‐regulation in *SKP2* expression when comparing cancer recurrence in PCa patients (Fig. 1F, *P* = 0.0459). Furthermore, patients who demonstrated invasion (bladder and seminal vesicle; Fig. S3a,b, *P* = 0.0473 and *P* = 0.0155, respectively) or progression from CRPC to NEPC (Fig. 1G, *P* = 0.0224) contained significantly up‐regulated levels of *SKP2*. Cell lines positive for Synaptophysin (SYP), a neuroendocrine PCa cell marker and thus measure of pro‐luminal phenotype and disease severity, contained higher SKP2 protein levels than cell lines not expressing SYP. These data suggest that up‐regulation of SKP2 plays an important role in PCa progression and severity (Fig. 1H). To validate our findings, we used castrated *Pten* ^*pc−/−*^; *Trp53* ^*pc−/−*^ mice undergoing regression or recurrence. IF staining revealed recurrent tumors in *Pten* ^*pc−/−*^; *Trp53* ^*pc−/−*^ mice demonstrated increased Syp and Skp2 protein levels compared to regressive tissues (Fig. S3c). Collectively, our results implicate SKP2–FOXA1 interactions through an inverse relation in pro‐luminal phenotype and disease severity.

### 3.2 *SKP2* knockdown increases FOXA1 protein levels in PCa cells

**Fig. 2:**
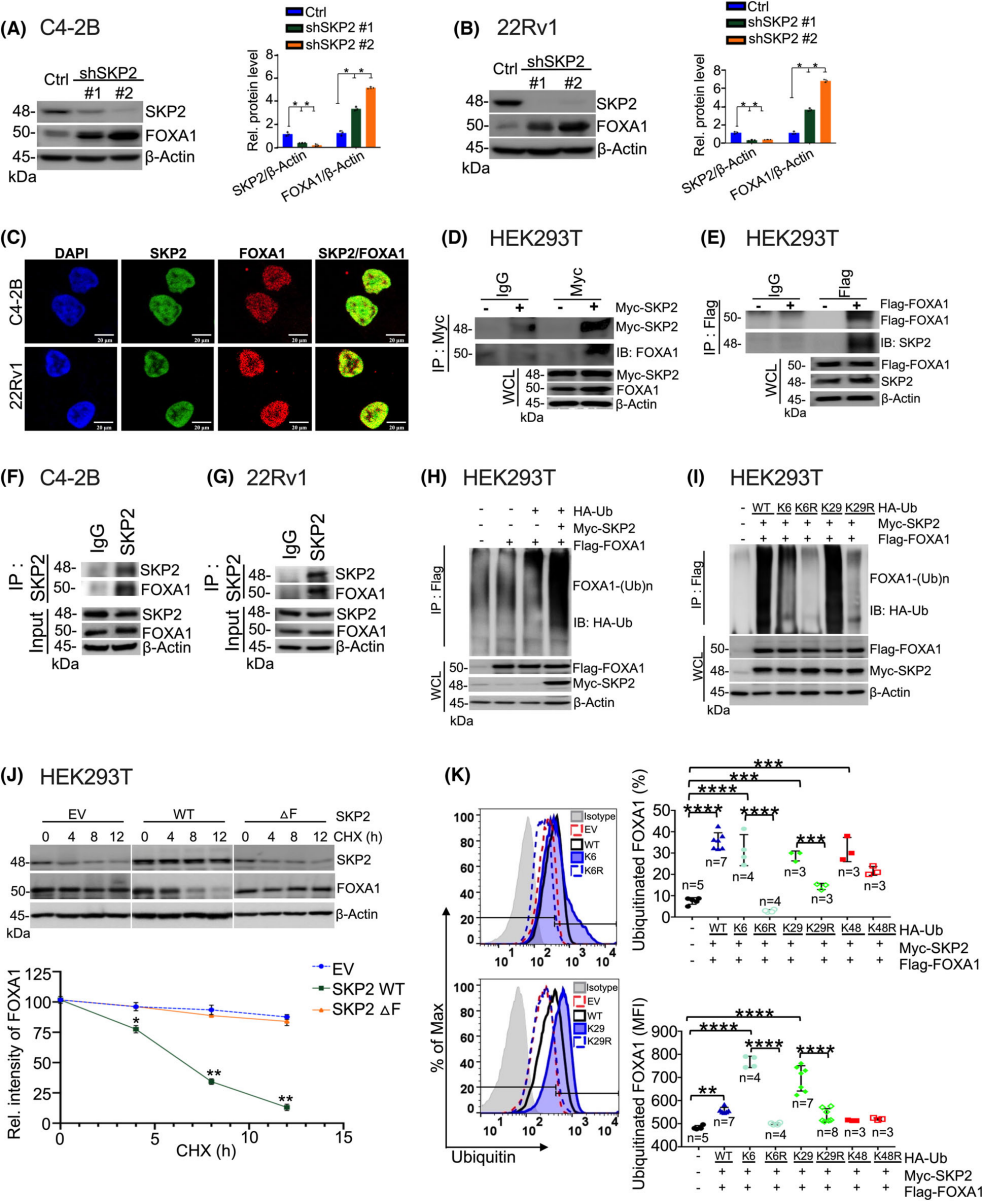
SKP2 regulates FOXA1 through K6 and K29‐linked ubiquitination. (A, B) Immunoblot analysis displays FOXA1 elevation in C4‐2B (*n* = 3) and 22Rv1 (*n* = 3) cells upon SKP2 knockdown (KD) via shRNA. Quantification analysis of the relative protein levels for SKP2 and FOXA1 is displayed on the right. (C) Immunofluorescence (IF) images demonstrate the colocalization of endogenous SKP2 and FOXA1 proteins in C4‐2B and 22Rv1 PCa cells. Scale bars are 20 μm. (D, E) Co‐immunoprecipitation analysis displays a physical interaction for SKP2 and FOXA1 proteins in HEK293T (*n* = 3) cells using Myc‐tagged SKP2 or Flag‐tagged FOXA1. WCL indicates the whole cell lysates. (F, G) Immunoprecipitation analysis displays an interaction for endogenous SKP2 and FOXA1 proteins in C4‐2B (*n* = 3) and 22Rv1 (*n* = 3) PCa cells. (H) *In vivo* ubiquitination assay displays an increase in HA‐Ub‐linked ubiquitination for FOXA1 by Myc‐SKP2 in HEK293T (*n* = 3) cells. (I) *In vivo* ubiquitination assay demonstrates a reduction in ubiquitination for K6R and K29R mutants (*n* = 3). (J) HEK293T cells were co‐transfected with Flag‐tagged FOXA1, HA‐ubiquitin (wild type, WT), and WT‐Myc‐SKP2 or a ▵F SKP2 mutant before being treated with Cycloheximide (CHX; 100 μg·mL^−1^) protein synthesis inhibitor for the indicated time points (h, hours). EV refers to empty vector. Lower panel is the corresponding plot for FOXA1 protein intensity (*n* = 3). (K) The percent and mean fluorescence intensity (MFI) of FOXA1 ubiquitination in HEK293T cells after overexpression of Myc‐SKP2, Flag‐FOXA1, and HA‐Ubiquitin WT or mutants. Colored points represent number of replicates per group. Comparisons between groups were analyzed using paired two‐tailed Student's *t*‐test. Bars indicate SEM. *P < 0.05, **P < 0.01, ***P < 0.001, ****P < 0.0001.

To investigate the type of relationship between SKP2 and FOXA1 in advanced PCa, we generated stable *SKP2* knockdown (KD) cell lines in C4‐2B and 22Rv1. These cell lines exhibit high SKP2 protein levels [27]. qRT‐PCR results confirmed effective *SKP2* KD in both C4‐2B and 22Rv1 cells (Fig. S4, *P* < 0.01). Immunoblot analysis further confirmed reduced SKP2 protein in each cell line (Fig. 2A,B). SKP2 KD significantly increased FOXA1 protein levels in both cell lines compared to the scrambled controls (Fig. 2A,B). Quantification of these results revealed that SKP2 KD resulted in a ~ 4‐fold increase of FOXA1 protein levels in C4‐2B cells and ~ 5‐fold increase of FOXA1 protein levels in 22Rv1 cells (Fig. 2A,B). These results suggest that SKP2 may have a regulatory effect on FOXA1 protein in advanced PCa.

### 3.3 Direct SKP2 and FOXA1 protein interactions in advanced PCa

To elucidate the type of physiological interaction between SKP2 and FOXA1 (direct or indirect) in advanced PCa, we first conducted immunofluorescence (IF) staining. Our results revealed nuclear colocalization of endogenous SKP2 and FOXA1 in both C4‐2B and 22Rv1 (Fig. 2C), suggesting close protein proximity and spatial overlap between SKP2 and FOXA1. To confirm the interaction, we overexpressed Myc‐SKP2 or Flag‐FOXA1 in HEK293T cells and performed co‐immunoprecipitation (co‐IP) for anti‐Myc or anti‐Flag antibodies. Immunoblot (IB) analysis showed that FOXA1 protein was detected in immunoprecipitates of Myc‐SKP2 compared to that with an empty vector (Fig. 2D), and SKP2 protein was also detected in the reciprocal immunoprecipitates of Flag‐FOXA1 (Fig. 2E). Therefore, we proceeded to evaluate the endogenous interaction of these two proteins in C4‐2B and 22Rv1 PCa cells. IP results indeed revealed protein–protein interactions between SKP2 and FOXA1 in both C4‐2B and 22Rv1 (Fig. 2F,G). Taken together, these results suggest a physical contact through binding interactions between SKP2 and FOXA1 in PCa.

### 3.4 FOXA1 is a substrate for SKP2‐mediated ubiquitination through K6‐ and K29‐linkages

To further assess SKP2‐FOXA1 protein interactions, we examined whether FOXA1 is a substrate for SKP2‐mediated degradation through ubiquitination. Therefore, we performed *in vivo* ubiquitination assay in HEK293T cells transfected with HA‐Ub, Myc‐SKP2, and Flag‐FOXA1 plasmids. As depicted, FOXA1 ubiquitination was strikingly increased with the addition of Myc‐SKP2 as compared to the controls (Fig. 2H). Specifically, the levels of FOXA1 ubiquitination were much lower in HEK293T cells when Myc‐SKP2 was absent (Fig. 2H), suggesting that SKP2 is required for FOXA1 ubiquitination.

Previous studies have reported that K48 is a common form of polyubiquitin linkage for protein degradation, and SKP2 has been reported to promote K48‐ and K63‐polyubiquitin chains [25]. For these reasons, we decided to identify the type of polyubiquitin linkage of FOXA1 mediated by SKP2. Seven ubiquitin (Ub) lysine (K) residues (K6, K11, K27, K29, K33, K48, and K63) have been identified to determine substrate fate on target proteins (e.g., lysosomal or proteasomal degradation). Transfecting these altered forms of ubiquitin into cells allow the identification of lysine linkages that are critical for determining protein fate (e.g., FOXA1). We, thus, co‐transfected HEK293T cells with Myc‐SKP2, Flag‐FOXA1, and HA‐Ub WT (wild‐type) plasmids or Ub mutant plasmids; where WT Ub included the full‐length ubiquitin sequence containing all lysine residues and K6, K11, K27, K29, K33, K48, or K63 included forms in which only the specified Ub lysine residue was available to form linkages. The addition of K48 or K63 Ub mutant plasmids resulted in a drastic reduction, instead of increase, in FOXA1 ubiquitination compared to HA‐Ub WT (Fig. S5a), indicating that neither K48 nor K63 plays a promoting role in the SKP2‐mediated polyubiquitination of FOXA1. Therefore, we turned our attention to the non‐canonical ubiquitin linkages at K6, K11, K27, K29, and K33. Interestingly, our results revealed that SKP2 promoted K6‐ and K29‐linked polyubiquitination of FOXA1 (Fig. S5b), and the levels of FOXA1 ubiquitination through K6‐ and K29‐linkage were comparable to that of WT‐Ub linkage (Fig. 2I). As expected, mutated lysine residues to arginine (R) at position 6 (K6R) or 29 (K29R) resulted in a dramatic decrease of FOXA1 polyubiquitination (Fig. 2I).

### 3.5 SKP2 determines the protein levels and stability of FOXA1 in human PCa cells

To test if SKP2 levels have any causal effects on FOXA1 ubiquitination, we first performed *in vivo* ubiquitination assay in HEK293T cells transfected with Flag‐FOXA1, HA‐Ub, and various doses of Myc‐SKP2 plasmids. As shown, SKP2 overexpression (OE) resulted in a significant increase of FOXA1 ubiquitination in HEK293T cells in a dose‐dependent manner, when compared to the controls (Fig. S6). Remarkably, ectopic expression of Myc‐SKP2 (*green*) resulted in a reduction of endogenous FOXA1 protein levels (*red*) in C4‐2B and 22Rv1 PCa cells, in contrast to adjacent non‐transfected cells (Fig. S7a).

We then proceeded to investigate if SKP2 overexpression would have a causal effect on the ubiquitination of FOXA1 in C4‐2B and 22Rv1 PCa cells. Our results demonstrated that overexpression of Myc‐SKP2 notably increased FOXA1 ubiquitination in C4‐2B and 22Rv1 PCa cells in a dose‐dependent manner as compared to the controls (Fig. S7b,c). Quantification analysis revealed a significant decrease in FOXA1 protein correlated with increases in SKP2 protein for C4‐2B and 22Rv1 cells (Fig. S7b,c). The results encouraged us to investigate if SKP2 KD would result in a contrasting effect on FOXA1 ubiquitination. To do so, we used stable SKP2 KD cells generated from C4‐2B and 22Rv1 cell lines (Fig. S4a,b). Our results showed that SKP2 KD resulted in a dramatic decrease of the endogenous FOXA1 ubiquitination in both C4‐2B and 22Rv1 (Fig. S8a,b) compared to the controls. Quantification analysis revealed a significant increase in FOXA1 protein levels correlated with SKP2 decrease in C4‐2B and 22Rv1 cells (Fig. S8a,b). In addition to the attenuated ubiquitination of FOXA1, SKP2 KD also prolonged the half‐life of FOXA1 protein in C4‐2B and 22Rv1 cells (Fig. S8c,d, *P* < 0.01). Collectively, these results suggest that FOXA1 levels are directly impacted by SKP2 through its E3 ligase activity.

To further understand the SKP2‐mediated regulation of FOXA1, we conducted cycloheximide chase experiments in HEK293T cells. Our results revealed that overexpression of SKP2‐WT significantly decreased the half‐life and protein levels of FOXA1, as compared to that from empty vector (EV) control and SKP2▵F mutant, which is unable to form a SKP2–SCF complex (Fig. 2J, *P* < 0.01). In addition, we also analyzed FOXA1 ubiquitination profiles in HEK293T cells using flow cytometry. A significant increase in the percentage and mean fluorescence intensity (MFI) of ubiquitinated FOXA1 was observed in HA‐Ub WT as compared to the vector controls (Fig. 2K, *P* < 0.0001). Remarkably, overexpression of Ub K6 and Ub K29 resulted in a significant increase in the MFI of FOXA1 ubiquitination, while K6R and K29R mutants significantly reduced FOXA1 ubiquitination MFI to vector control levels (Fig. 2K, *P* < 0.001). These results further suggest that K6‐ and K29‐linked ubiquitination are essential for the ubiquitination of FOXA1.

### 3.6 Skp2 knockout increases Foxa1 levels independent of Ar signaling in mouse models

**Fig. 3:**
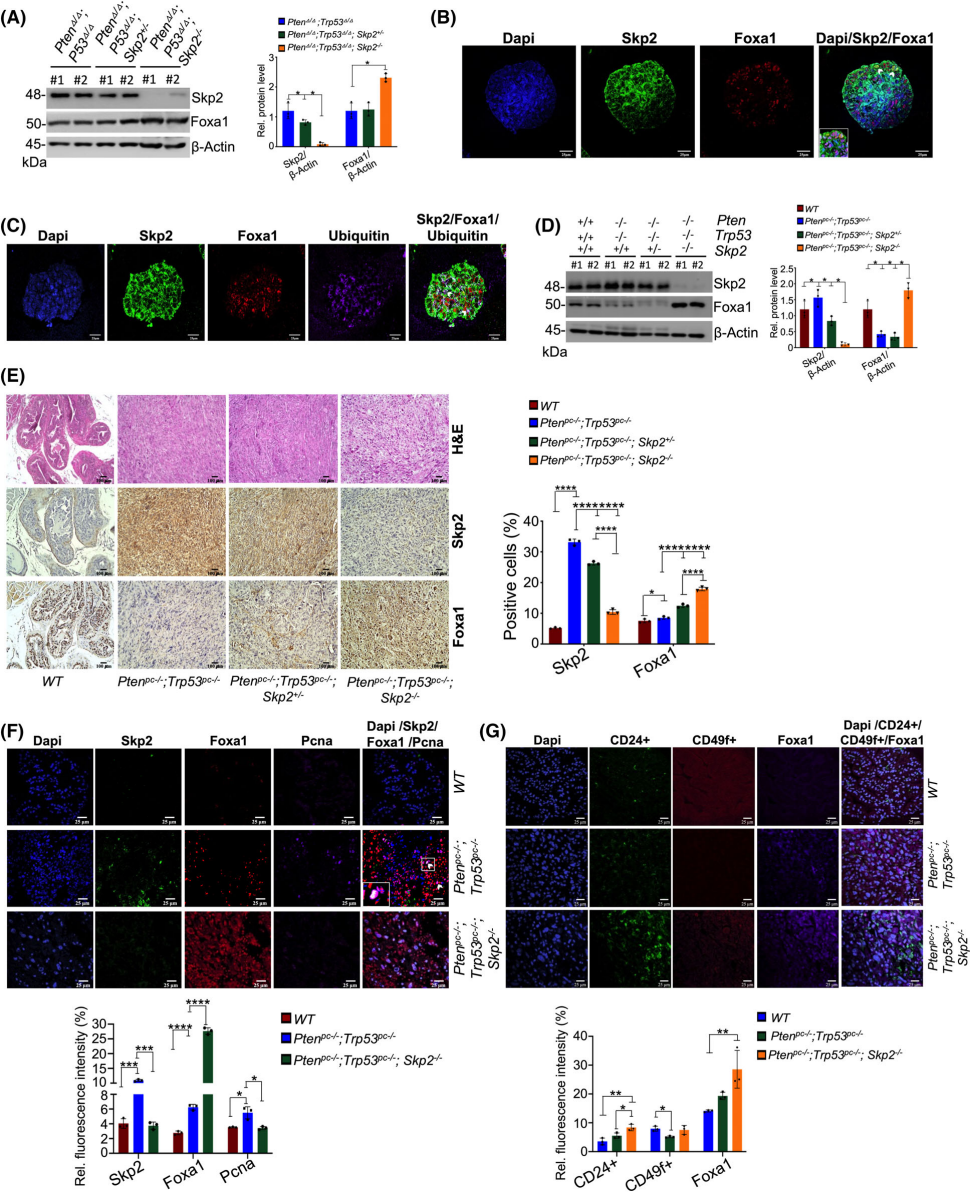
Skp2 Abrogation elevates Foxa1 levels *in vivo* in mouse models. (A) Immunoblot analysis shows the effects of Skp2 on Foxa1 protein levels in mouse embryonic fibroblasts (MEFs) with indicated genotypes. Quantification of protein levels is relative to beta‐Actin and displayed in the right panel (*n* = 3). (B, C) Immunofluorescence (IF) images show colocalization among Skp2, Foxa1, and ubiquitin in murine prostate organoids (*n* = 3). White arrows indicate colocalization for Skp2, Foxa1, and Ubiquitin. Scale bars are 25 μm. (D) Western blotting analysis of Skp2 and Foxa1 protein levels for anterior prostate (AP) tissues of mice with the indicated genotypes. Quantification analysis for the corresponding protein expression is shown in the right panel (*n* = 3). (E) Immunohistochemistry (IHC) staining of Skp2 and Foxa1 in prostate tissues of mice with the indicated genotypes. The side panel displays a quantification analysis for IHC staining (*n* = 3). Scale bars are 100 μm. (F) IF images show colocalization (white arrows) among Skp2, Foxa1, and Pcna (proliferating cell nuclear antigen) prostate tissues in *Pten* ^*pc−/−*^; *Trp53* ^*pc−/−*^ mutant mice, while Skp2 loss in *Pten* ^*pc−/−*^; *Trp53* ^*pc−/−*^; *Skp2* ^*−/−*^ mutant mice has decreased Skp2, Foxa1, and Pcna colocalization (*n* = 3). Scale bars are 25 μm. (G) IF images for luminal (CD24^+^) and basal (CD49f^+^) lineage markers in *Pten* ^*pc−/−*^; *Trp53* ^*pc−/−*^ and *Pten* ^*pc−/−*^; *Trp53* ^*pc−/−*^; *Skp2* ^*−/−*^ mutant mice (*n* = 3). Scale bars are 25 μm. Comparison between groups was performed using Student's *t*‐test. Bars indicate SEM. *P < 0.05, **P < 0.01, ***P < 0.001, ****P < 0.0001.

Literature demonstrated that combined inactivation of PTEN and TP53 promotes PCa progression [35]. Elevated SKP2 levels were also associated with PTEN loss resulting in poor patient outcome and PCa progression, while reduced SKP2 has been shown to decrease prostate tumorigenesis [25, 36, 37]. We, therefore, took advantage of our *Pten/Trp53/Skp2* triple‐null (*Pten* ^*Δ/Δ*^; *Trp53* ^*Δ/Δ*^; *Skp2* ^*−/−*^) mouse embryonic fibroblasts (MEFs), which do not express Ar signaling to investigate if Skp2‐mediated regulation of Foxa1 was Ar independent. Our results showed that *Skp2* knockout (KO) led to a significant ~2‐fold increase in Foxa1 levels in *Pten/Trp53/Skp2* triple‐null (*Pten* ^*Δ/Δ*^; *Trp53* ^*Δ/Δ*^; *Skp2* ^*−/−*^) MEFs as compared to that in *Pten/Trp53* (*Pten* ^*Δ/Δ*^; *Trp53* ^*Δ/Δ*^) MEFs (Fig. 3A). However, inactivation of one allele for the *Skp2* gene in MEFs (*Pten* ^*Δ/Δ*^; *Trp53* ^*Δ/Δ*^; *Skp2* ^*+/−*^) slightly reduced Skp2 protein while the levels of Foxa1 remained unaffected (Fig. 3A). Having documented the SKP2–FOXA1 interplay in human PCa and MEFs, we next assessed whether this interplay was consistent in models including malignant prostate epithelial cells. Thus, we evaluated Skp2–Foxa1 interactions within murine PCa organoids. Analogous to our previous results, IF revealed enhanced ubiquitin intensity in areas of Skp2–Foxa1 nuclear colocalization at week 4 of organoid passage (Fig. 3B,C, white arrows). Week 4 organoids demonstrated increased staining for non‐luminal cells (CD49f^+^; Fig. S9a). In contrast, CD24^+^ cells remained relatively unaffected compared to week 1 organoids (Fig. S9a). Since Foxa1 is a key driver in luminal lineage, these data suggest that Skp2 may be promoting non‐luminal lineage markers through Foxa1 protein regulation.

We then proceeded to investigate the effects of Skp2 KO in the ubiquitin‐mediated regulation of Foxa1 in prostate tumors of conditional knockout (pc^−/−^) *Pten* ^*pc−/−*^; *Trp53* ^*pc−/−*^; *Skp2* ^*−/−*^ mutant mice. *Pten* ^*pc−/−*^; *Trp53* ^*pc−/−*^ conditional double‐null mice are representative of advanced PCa, with mice developing prostate tumors as early as 3 months of age [23]. WB analysis of prostate tissues showed Foxa1 protein levels increased ~ 4‐fold in *Pten* ^*pc−/−*^; *Trp53* ^*pc−/−*^; *Skp2* ^*−/−*^ mice as compared to that in *Pten* ^*pc−/−*^; *Trp53* ^*pc−/−*^ mice (Fig. 3D). Akin to what was observed in advanced PCa human specimens, IHC staining revealed a reverse correlation between Foxa1 and Skp2 protein levels in normal prostates of WT mice and *Pten* ^*pc−/−*^; *Trp53* ^*pc−/−*^ mutant mice (Fig. 3E). However, Skp2 loss significantly increased Foxa1 levels in prostate tumors of *Pten* ^*pc−/−*^; *Trp53* ^*pc−/−*^; *Skp2* ^*+/−*^ and *Pten* ^*pc−/−*^; *Trp53* ^*pc−/−*^; *Skp2* ^*−/−*^ mice in a dose‐dependent manner, indicating an essential role for Skp2 in Foxa1 regulation (Fig. 3E; Fig. S9b–d). Quantification analysis revealed that Foxa1 levels increased ~ 2.9‐fold in *Pten* ^*pc−/−*^; *Trp53* ^*pc−/−*^; *Skp2* ^*−/−*^ mice compared to *Pten* ^*pc−/−*^; *Trp53* ^*pc−/−*^ mice (Fig. 3E). Worthy of note, Skp2 ablation with concurrent enhanced levels of Foxa1 in *Pten* ^*pc−/−*^; *Trp53* ^*pc−/−*^; *Skp2* ^*−/−*^ mice resulted in decreased tumor burden by suppressing prostate tumor proliferation. Specifically, immunofluorescence (IF) imaging demonstrated that Skp2 loss reduced the levels of Pcna, a nuclear proliferation marker, in *Pten* ^*pc−/−*^; *Trp53* ^*pc−/−*^; *Skp2* ^*−/−*^ as compared to *Pten* ^*pc−/−*^; *Trp53* ^*pc−/−*^ mice (Fig. 3F). Importantly, Foxa1 (*red*) and Skp2 (*green*) colocalized with Pcna (*purple*) in *Pten* ^*pc−/−*^; *Trp53* ^*pc−/−*^ mice while the prostates of WT and *Pten* ^*pc−/−*^; *Trp53* ^*pc−/−*^; *Skp2* ^*−/−*^ mice displayed reduced amounts of Foxa1, Skp2, and Pcna colocalization (Fig. 3F). Analogous to what was observed in advanced human PCa tissues, Skp2:Foxa1 ratios were enhanced in *Pten* ^*pc−/−*^; *Trp53* ^*pc−/−*^ compared to both WT (*P* < 0.0001) and *Pten* ^*pc−/−*^; *Trp53* ^*pc−/−*^; *Skp2* ^*−/−*^ (*P* < 0.0001) mice (Fig. S9e), once again stressing the relevance of Skp2–Foxa1 interplay in the advancement of PCa. We used both basal (CD49f^+^) and luminal (CD24^+^) markers to visualize changes in cell lineage upon Skp2 KO. Skp2 loss increased CD24^+^ luminal cells simultaneously with enhanced Foxa1 levels in *Pten* ^*pc−/−*^; *Trp53* ^*pc−/−*^; *Skp2* ^*−/−*^ mice compared to *Pten* ^*pc−/−*^; *Trp53* ^*pc−/−*^ (Fig. 3G). In contrast, basal (CD49f^+^) positive cells were significantly decreased in *Pten* ^*pc−/−*^; *Trp53* ^*pc−/−*^ compared to *WT* mice and remained consistent in *Pten* ^*pc−/−*^; *Trp53* ^*pc−/−*^; *Skp2* ^*−/−*^ mice (Fig. 3G). These findings indicate that Skp2 ablation results in a striking effect on Foxa1 protein levels (independent of Ar signaling), providing critical evidence for the significance of the ubiquitin‐mediated degradation of Foxa1 by Skp2.

### 3.7 SKP2 pharmaceutical inhibition decreases SKP2:FOXA1 Ratio and tumor proliferation in nude mice

**Fig. 4:**
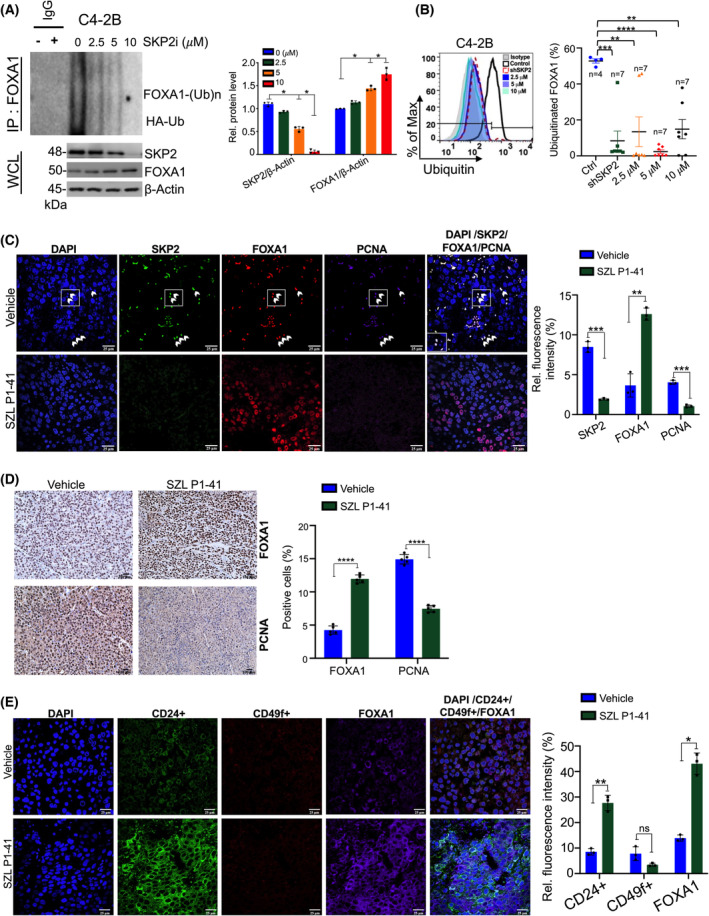
SKP2 alters FOXA1 ubiquitination levels in human prostate cancer cells in a dose‐dependent manner. (A) Endogenous ubiquitination of FOXA1 in C4‐2B proceeding SKP2 inhibition using SZL P1‐41 at different concentrations (μm). Samples were collected and subjected to *in vivo* ubiquitination and western blot analysis (*n* = 3). (B) FOXA1 ubiquitination profile using flow cytometry for vehicle, SKP2 KD, and SKP2 inhibition using SZL P1‐41 for C4‐2B. Representative histogram and percent ubiquitination of FOXA1 are plotted. Colored points represent number of replicates per group. (C) C4‐2B xenograft mice received vehicle (DMSO) or SKP2 inhibitor SZL P1‐41 (30 mg·kg^−1^; three times per week, intraperitoneal, i.p. injection) for 30 days. Immunofluorescence (IF) staining displays increased colocalization (white arrows) amongst SKP2, FOXA1, and PCNA for C4‐2B xenograft vehicle tissues, while exposure to SZL P1‐41 treatment reduced SKP2 and PCNA levels (*n* = 3). Scale bars are 25 μm. (D) C4‐2B xenograft tissue sections were subjected to Immunohistochemistry (IHC) staining with the indicated antibodies (*n* = 5). Scale bars are 100 μm. (E) IF luminal (CD24^+^) and basal (CD49f^+^) lineage staining in C4‐2B vehicle and SZL P1‐41‐treated mice (*n* = 3). Scale bars are 25 μm. Comparison between groups was performed using Student's *t*‐test. Bars indicate SEM. *P < 0.05, **P < 0.01, ***P < 0.001, ****P < 0.0001.

To examine whether SKP2–FOXA1 interplay could be targeted using SKP2 pharmaceutical inhibition in advanced PCa, we implanted C4‐2B and 22Rv1 cells subcutaneously into the dorsal flanks of nude mice. Prior to doing so, we first confirmed analogous results to KD and KO findings. SZL P1‐41, a compound that targets SKP2 E3 ubiquitin ligase activity [18], resulted in a significant decrease of FOXA1 ubiquitination coupled with an increase in FOXA1 protein in C4‐2B and 22Rv1 cells (Fig. 4A; Fig. S10). Profile analysis revealed that SKP2 KD and SZL P1‐41 treatment in C4‐2B and 22Rv1 resulted in significant declines in the percent of ubiquitinated FOXA1, but not MFI (Fig. 4B; Fig. S11a,b). We randomly divided mice into cohorts treated with either a vehicle (DMSO) control or 30 mg·kg^−1^ of SZL P1‐41 three times weekly. IF staining demonstrated increased amounts of PCNA (*purple*) in vehicle‐treated controls, suggesting enhanced PCa cell growth in areas of increased SKP2 (*green*) and FOXA1(*red*) protein staining (Fig. 4C; Fig. S12a), in contrast to SZL P1‐41‐treated mice which demonstrated decreased PCNA upon SKP2 inhibition (Fig. 4C; Fig. S12a). SKP2:FOXA1 ratios were also significantly reduced following SZL P1‐41 treatment, confirming effective administration and validating a significant decline in SKP2 protein levels after inhibition (Fig. S12b,c). Reduction in SKP2:FOXA1 indicates that pharmaceutical inhibition of SKP2 is sufficient to target the SKP2–FOXA1 interplay and subsequently increase FOXA1 protein levels, while simultaneously suppressing tumor proliferation (Fig. S12a–c). A similar pattern of IHC staining for PCNA and FOXA1 protein levels was observed upon SKP2 inhibition, suggesting that SKP2 regulation of FOXA1 may, in turn, drive tumor proliferation and serve as *in vivo* validation for our *in vitro* findings (Fig. 4D; Fig. S12d). Moreover, SZL P1‐41‐treated nude mice corroborated increases in CD24^+^ luminal cells with increases in FOXA1 protein levels, while CD49f^+^ cells had no significant differences (Fig. 4E; Fig. S12e). These data suggest that pharmacological inhibition of SKP2 results in increased FOXA1 levels, promoting a luminal‐like identity.

### 3.8 SKP2‐mediated ubiquitination of FOXA1 occurs at the C‐terminal TAD

To delineate the subdomains for FOXA1 ubiquitination, we performed *in vivo* ubiquitination assays using seven truncated FOXA1 mutant plasmids including the N‐terminal and C‐terminal transactivating domain (TAD) and/or the forkhead domain (FKHD; Fig. S13a). Our results demonstrated that the ubiquitination of truncated FOXA1 1–385 amino acids(aa) or 59‐345aa was dramatically decreased as compared to that of FOXA1 WT, suggesting the importance of the C‐terminal TAD (C‐TAD) in the conjugation of FOXA1 polyubiquitination (Fig. S13b). In agreement with that, *in vitro* co‐IP assays for domain mapping displayed a stronger SKP2‐FOXA1 interaction for the truncated FOXA1 141–466 aa or 295–466 aa found within the FOXA1 C‐TAD upon SKP2 pull‐down (Fig. S13c).

To define the potential ubiquitination sites for FOXA1 by SKP2, we searched for the candidate K residues of FOXA1 at UbPred (www.ubpred.org). K414 of FOXA1 within the C‐TAD was predicted with high confidence to be the most likely ubiquitinated residue (Fig. S14). Therefore, we generated FOXA1 mutant plasmids, and our results demonstrated FOXA1 mutation at K414A or K418A resulted in a noticeable decrease of the SKP2‐mediated ubiquitination of FOXA1 as compared to other FOXA1 mutants, suggesting that both K414 and K418 in FOXA1 may be important sites for polyubiquitination (Fig. S15a). However, a double mutation with K414A/K418A reduced but failed to abolish SKP2‐mediated ubiquitination of FOXA1. Profile analysis by flow cytometry displayed an increase in the percentage of ubiquitinated FOXA1 when overexpressing Myc‐SKP2 and FOXA1 WT in HEK293T cells (Fig. S15b, *P* < 0.0001). Remarkably, overexpression of Myc‐SKP2 or the truncated FOXA1 295‐466aa of the C‐TAD region displayed a higher percentage in FOXA1 ubiquitination as compared to the vector controls (Fig. S15b, *P* < 0.0001), whereas the percent and the MFI of FOXA1 ubiquitination were drastically decreased for K414A and K418A mutants, respectively (Fig. S15b, *P* < 0.001) compared to the controls. These results suggest that K414 and K418 residues in the C‐TAD play an important role in the SKP2‐mediated ubiquitination of FOXA1.

### 3.9 SKP2 facilitates FOXA1 degradation through lysosomal‐dependent pathways

**Fig. 5:**
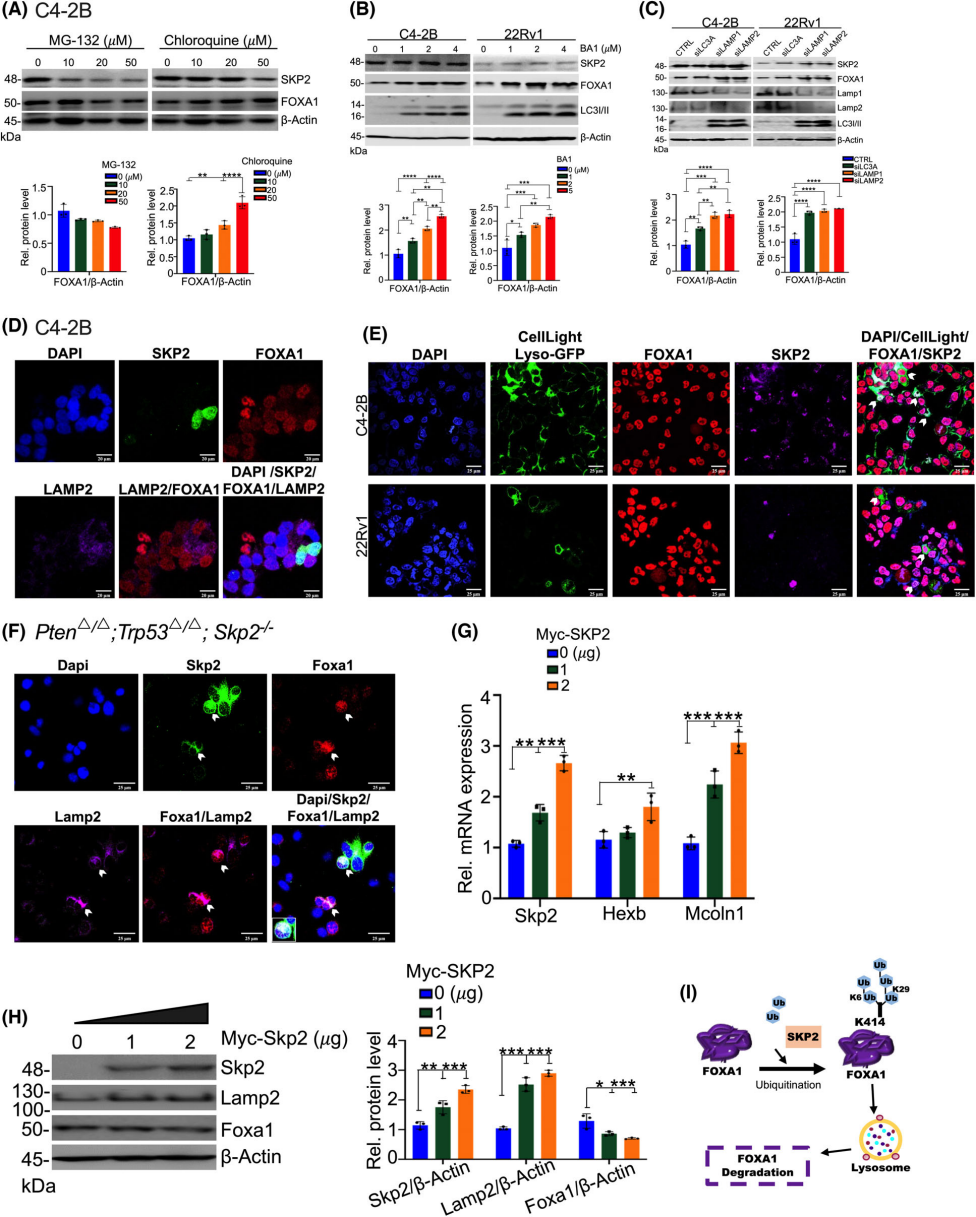
FOXA1 Regulation by SKP2 is a lysosomal‐dependent event. (A) The effects of Chloroquine on FOXA1 levels in C4‐2B cells. Cells were subjected to MG132 or chloroquine treatment at the indicated concentrations (μm). Lower panels display the quantification for FOXA1 protein levels (*n* = 3). (B) The effects of bafilomycin A1 (BA1) treatment at the indicated concentrations (μm) on FOXA1 protein levels in C4‐2B and 22Rv1 cells. Quantification for FOXA1 levels is displayed below (*n* = 3). (C) Immunoblot demonstrating the effects of lysosomal marker (LC3, LAMP1, and LAMP2) KD on FOXA1 protein levels in C4‐2B and 22Rv1 cells. Lower panels demonstrate quantified FOXA1 protein levels (*n* = 3). (D) Immunofluorescence (IF) images display an increase in LAMP2 (lysosome‐associated membrane glycoprotein) levels in C4‐2B cells upon Myc‐SKP2 overexpression (*n* = 3). Scale bars are 20 μm. (E) CellLight Lysosome‐GFP, BacMam 2.0, labeling of lysosomal activity in association with FOXA1 in C4‐2B (*n* = 3) and 22Rv1 (*n* = 3). White arrows indicate colocalization between the lysosome and FOXA1. Scale bars are 25 μm. (F) IF images show the impact of Skp2 restoration on Lamp2 in *Pten/Trp53/Skp2* triple‐null mouse embryonic fibroblasts (MEFs) proceeding transient overexpression of Myc‐SKP2 (*n* = 3). White arrows indicate colocalization amongst Lamp2, FOXA1, and Skp2. Scale bars are 25 μm. (G) qRT‐PCR analysis of Skp2 and the lysosomal genes Hexb and Mcoln1 in *Pten/Trp53/Skp2* triple‐null MEFs proceeding the transient overexpression of Myc‐SKP2 or an empty vector (EV). Results represent relative expression values to the housekeeping gene beta‐Actin (*n* = 3). (H) Western blotting analysis of Skp2, FOXA1, and Lamp2 levels for *Pten/Trp53/Skp2* triple‐null MEFs proceeding overexpression of Myc‐SKP2 (0–2 μg). Quantification analysis for the corresponding relative protein levels is shown in the right panel (*n* = 3). (I) A working model on the K6‐ and K29‐linked ubiquitination of FOXA1 by SKP2 in lysosomal degradation. Comparison between groups was performed using Student's *t*‐test. Bars indicate SEM. *P < 0.05, **P < 0.01, ***P < 0.001, ****P < 0.0001.

To investigate the major polyubiquitin‐mediated degradation pathways, we assessed FOXA1 levels in PCa cells after a 4‐h treatment with either MG‐132 (an inhibitor of the proteasomal degradation) or chloroquine (an inhibitor for the lysosomal degradation). Chloroquine treatment at 50 μm resulted in a significant increase in FOXA1 protein levels in C4‐2B and 22Rv1 cells, whereas MG‐132 treatment at 50 μm decreased FOXA1 levels (Fig. 5A; Fig. S16). Similarly, chloroquine treatment also increased the protein levels of FOXA1 in HEK293T cells transfected with HA‐Ub, Myc‐SKP2, and Flag‐FOXA1 (Fig. S17). To further confirm that SKP2 promotes FOXA1 degradation via a lysosome‐dependent mechanism, we assessed FOXA1 protein levels in C4‐2B and 22Rv1 cells after Bafilomycin A1 (BA1) treatment, an inhibitor of lysosomal degradation and vacuolar H^+^‐ATPases. BA1 treatment at 1, 2, and 5 μm significantly increased FOXA1 protein levels in C4‐2B and 22Rv1 cells in a dose‐dependent manner, while SKP2 protein levels were marginally affected (Fig. 5B). These results strongly suggest that increased FOXA1 protein levels are due to inhibition of lysosomal function. We also used siRNA to silence several lysosomal markers important in maintaining lysosome membrane integrity including LC3, LAMP1, and LAMP2. Our results revealed increases in FOXA1 protein upon KD of each lysosomal protein marker in C4‐2B and 22Rv1 cells (Fig. 5C), suggesting that SKP2‐mediated ubiquitination of FOXA1 promotes degradation of FOXA1 through a lysosomal‐dependent pathway. Notably, IF imaging demonstrated that ectopic expression of Myc‐SKP2 reduced FOXA1 levels through recruitment of lysosomes. Along with the reduced amounts of FOXA1, SKP2 expression (*green*) colocalized with the lysosome marker LAMP2 (*purple*) in C4‐2B and 22Rv1 PCa cells (Fig. 5D; Fig. S18). GFP labeling of lysosomes in live C4‐2B and 22Rv1 cells using CellLight Lysosome‐GFP, BacMam 2.0 further indicated recruitment of lysosome‐GFP within areas that also stained positively for FOXA1 protein (Fig.5e), suggesting lysosomal activity in regions of FOXA1 within C4‐2B and 22Rv1 cells.

To confirm that Skp2‐mediated ubiquitination of Foxa1 was through lysosomal recruitment, we enforcedly expressed Skp2 in *Pten/Trp53/Skp2* (*Pten* ^*Δ/Δ*^; *Trp53* ^*Δ/Δ*^; *Skp2* ^*−/−*^) triple‐null MEFs (Fig. S19). Consistent with our findings in human PCa cells, IF staining revealed that Skp2 restoration dramatically increased Lamp2 levels, indicating lysosomal recruitment and localization (Fig. 5F). To further assess the significance of these findings, we asked ourselves whether Skp2 restoration increased Lamp2 and lysosome‐associated genes in a dose‐dependent manner. As confirmed, *Skp2* mRNA levels were significantly increased in transfected MEFs compared to vector controls, and Skp2 restoration increased the mRNA abundance of lysosomal genes *Hexb* and *Mcoln1* in *Pten/Trp53/Skp2* triple‐null MEFs in a dose‐dependent manner (Fig. 5G). Immunoblot analysis further confirmed that Skp2 restoration significantly increased Lamp2 protein levels in *Pten/Trp53/Skp2* triple‐null MEFs (Fig. 5H), while the stability and half‐life of Foxa1 protein was prolonged in *Pten/Trp53/Skp2* triple‐null MEFs compared to *Pten/Trp53* (*Pten* ^*Δ/Δ*^; *Trp53* ^*Δ/Δ*^) double‐null MEFs (Fig. S20). A summary of our findings is illustrated in Fig. 5I.

## 4 Discussion

Targeting FOXA1 remains clinically challenging despite its crucial role in the development and progression of PCa. In addition, FOXA1 protein levels vary greatly based on PCa severity [38], making it difficult to target, but the reasons have not been clearly identified. In the present study, we revealed several unexpected findings which serve to clarify the role of FOXA1 in PCa progression. Our data provided evidence indicating that the interplay between SKP2 and FOXA1, and not just SKP2 or FOXA1 levels alone, are better indicators of PCa prognosis, highlighting the significance of SKP2–FOXA1 interactions as a potential biomarker for severe PCa. In addition, our findings suggest that targeting the SKP2–FOXA1 interplay through SKP2 inhibition during advanced stages of PCa is more beneficial than targeting these interactions during earlier stages for restoring luminal positive cells, suggesting a possible clinical application.

FOXA1 levels vary across advanced PCa, yet our understanding of the regulatory mechanisms involved has not been clearly identified. Emerging evidence revealed that aberrant levels and mutations of FOXA1 are associated with castration‐resistant PCa (CRPC) and neuroendocrine PCa (NEPC) malignancies. Studies from several groups demonstrated that reduction of FOXA1 levels is found in human NEPC samples and FOXA1 KD or KO in LNCaP cells promotes the NEPC phenotype [15, 16]. These studies suggest that FOXA1 loss may not be beneficial in advanced stages of PCa. As FOXA1 remains crucial in promoting neuroendocrine differentiation [15], identifying the most effective prognostic markers and therapeutic strategies for NEPC is vital. Therefore, targeting the strong link between SKP2‐FOXA1 may prove to be beneficial in this context. In contrast, FOXA1 levels are frequently up‐regulated in human‐localized PCa and CRPC, suggesting seemingly opposing mechanisms. Our findings revealed increased SKP2 expression in recurrent PCa and NEPC (Fig. 1F,G; Fig. S3c) as well as increased SKP2:FOXA1 protein ratios at stage IV PCa in human prostate TMA (Fig. 1C–E; Fig. S2). SKP2:FOXA1 ratios assess the intensity values for SKP2 and FOXA1 in relation to each other and in relevance to the stage of PCa, thus providing a potential biomarker for predicting PCa progression and positive treatment responses to SKP2 therapy. According to tissue microarray (TMA) data, patients classified with stage II PCa (localized tumor with medium to low PSA levels) had comparable SKP2:FOXA1 ratios to normal prostate, indicating similar protein levels and no significant differences in abundance. In contrast, stage III (locally advanced PCa with high PSA) showed significantly lower SKP2:FOXA1 ratios than normal prostate tissue, thus indicating the levels of FOXA1 increased from stage II to stage III PCa with a concurrent increase in SKP2. These findings imply that SKP2 treatment at stage II or III would be counterproductive. Noteworthy, stage IV PCa (where PCa spreads beyond the borders of the prostate) indicated a significant enhancement of SKP2:FOXA1 ratios compared to normal prostate and stage II and III PCa, suggesting elevated SKP2 protein with concurrent declines in FOXA1 and implying that SKP2 inhibition would be beneficial in these patients. These findings were further validated *in vivo* where C4‐2B and 22Rv1 xenograft nude mice with elevated SKP2:FOXA1 ratios in vehicle controls consistently showed significantly reduced PCNA levels in response to SZL P1‐41 treatment. Moreover, SZL P1‐41 treatment increased FOXA1 levels as well as luminal marker CD24^+^. This underscores the significance of targeting the SKP2‐FOXA1 interplay as cells expressing luminal lineage demonstrate sensitivity to antiandrogen therapies while non‐luminal phenotypes frequently demonstrate antiandrogen resistance [26]. Collectively, these data suggest that it is the protein interactions that exist between SKP2 and FOXA1 that drive PCa progression, which was previously unknown.

Increased SKP2 levels are associated with the development and progression of various human cancers, including PCa [39, 40, 41]. We previously demonstrated that SKP2 is involved in the regulation of AR in PCa cells [27]. Given the pivotal role of the FOXA1/AR coupling in PCa, our new findings suggest that SKP2 regulates the FOXA1/AR axis and their downstream targets. The interesting scenario is that SKP2 executes its E3 ligase function for FOXA1 (in lysosome) and AR (in proteasome) in PCa cells in a well‐coordinated manner. Thus, it is likely the SKP2–FOXA1 interplay may promote the SKP2‐AR cascade, and not vice versa, since the SKP2–FOXA1 interaction occurs in both AR^+^ cells (C4‐2B and 22Rv1) and AR‐ MEFs (Fig. 5F–H).

Post‐translational modifications (PTM), such as ubiquitination, are associated with PCa progression [42, 43]. E3 ubiquitin (Ub) ligases like SKP2 covalently link the carboxy terminus of the ubiquitin peptide to the amino group of a lysine (K) residue on the target protein to determine its fate. Therefore, identifying the ubiquitination site(s) of target proteins and the type of polyubiquitin linkage is critical for the development of effective therapeutic strategies, as defects in these have been associated with increased cancer risk [44, 45, 46]. Our current study revealed that SKP2 selectively engaged in K6‐ and K29‐linked polyubiquitination, a previously uncharacterized polyubiquitin signal for SKP2, to drive lysosomal degradation of FOXA1 (Fig. 2H,I). K29‐linked ubiquitin chains have been implicated in the degradation processes of target proteins in conjunction with other canonical and non‐canonical ubiquitin linkages [47, 48, 49]. For example, in conjunction with the canonical ubiquitin K48‐linkage, proteins are subject to degradation through a proteasomal‐dependent machinery [50]. Emerging evidence revealed that the lysosome is also involved in ubiquitin‐mediated degradation [51]. Previous studies have also reported that K29‐ and K63‐linked ubiquitination can promote lysosomal degradation of substrates including transmembrane proteins like the LDL receptor and nuclear proteins such as HIF1A [52, 53, 54]. K29‐linked ubiquitination was shown to promote lysosomal‐mediated degradation [55, 56], supporting our findings that K6‐ and K29‐linked ubiquitination promote lysosomal degradation of FOXA1 (Fig. 5I). Several reports have also associated K6‐linked ubiquitin chains with the lysosome but with limited understanding [56, 57, 58]. For example, HUWE1, a HECT domain‐containing E3 ligase, is reported to promote the protein degradation of its substrates by catalyzing K6‐ubiquitination in conjunction with K11 and K48 [59]. Interestingly, Ub‐K6R mutation almost abolished the signals (percent and MFI) of FOXA1 ubiquitination (Fig. 2K) compared to Ub‐K29R mutation, suggesting that K6‐ and K29‐linked polyubiquitination may not play equivalent roles in the SKP2‐mediated polyubiquitination of FOXA1 in PCa. Various types of ubiquitin linkages are substrate specific and rely heavily on the affinity between E3 ligase and substrate [60]. Non‐canonical polyubiquitin chains (such as K6 and K29) constitute an important part of ubiquitination systems which assist in the control and determination of many cellular processes including PCa progression [61]. Nonetheless, we cannot rule out the possibility that other pathways associated with E3 ligase function may also be involved in regulating FOXA1 levels in PCa. In human gastric cancer, down‐regulation of FOXA1 by the E3 ligase ZFP91 has been associated with promotion of chemoresistance [62], while in colon cancer down‐regulation of FOXA1 via NEDD4 promoted EMT and metastasis [63]. These publications support the possibility that FOXA1 regulation is cancer and cell‐type specific. In PCa, EZH2‐regulated deubiquitination of FOXA1 via USP7 promotes FOXA1 protein stability, suggesting that patients with high FOXA1 expression may have greater sensitivity to targeted therapies [64]. Hence, it is essential that future studies continue to unravel the mechanisms of FOXA1 regulation at each stage in PCa and within the context of treatment response in order to attenuate PCa severity.

Furthermore, we identified K414 and K418 in the C‐terminal transactivating domain (C‐TAD) of FOXA1 as significant conjugation sites for SKP2‐mediated ubiquitination. The C‐TAD domain of FOXA1 contributes to chromatin binding and DNA affinity by increasing FOXA1 binding to the KLK3 enhancer element [65, 66]. Mutations in the C‐TAD region of FOXA1 are associated with increased activity and PCa progression [67], highlighting the significance of our findings. It is possible that the concurrent enhancement of SKP2 and FOXA1 protein levels typically detected in PCa is due to mutations in the C‐TAD domain of FOXA1 which may affect SKP2‐recognition and degradation of FOXA1. Studies in PCa patients revealed that the C‐terminal region of FOXA1 bears many point mutations. It remains to be determined whether SKP2‐mediated ubiquitination of FOXA1 is also affected by FOXA1 C‐TAD mutations contributing to the activation of oncogenic pathways. Individual mutations for K414A and K418A resulted in significant decreases in FOXA1 ubiquitination (Fig. S15a,b). However, combined K414 and K418 mutations decreased but failed to completely abolish FOXA1 ubiquitination (Fig. S15a). Our study identified several lysine residues in FOXA1 that are recognized for ubiquitination (Fig. S14). Thus, it is possible that upon simultaneous unavailability of K414 and K418, SKP2 alternatively promotes the ubiquitin conjugation of other lysine residues. Supporting this concept, truncation of C‐TAD FOXA1 (1–385) decreased SKP2 ubiquitination, while further truncation (1–294) retained SKP2 ubiquitination. We hypothesize that specific FOXA1 truncations (e.g., 1–294) may fold in a manner that is akin to the WT protein, allowing for retention of surface charges that may expose key lysine residues (K6 and K288) that are recognized by SKP2, whereas, these key lysine residues may become occluded in other FOXA1 truncations (e.g., 1–385) due to changes in the 3D structure of FOXA1. Previous studies have reported that E3 ligases such as Mdm2 utilize allosteric recognition, including protein structure, surface charges, and protein stability, to mediate the degradation of truncated isoforms [68]. Future studies are needed to explore the biological effects of C‐TAD truncations and mutations on the SKP2‐mediated ubiquitination of FOXA1 in PCa. Nonetheless, our findings suggest that the SKP2–FOXA1 interplay is a better prognosis factor for PCa and merits further investigation into promoting a more luminal‐like identity which may enhance therapeutic sensitivity to abrogate FOXA1‐driven PCa.

## 5 Conclusions

FOXA1 is a transcriptional activator for steroid hormone receptors that can promote luminal epithelial cell differentiation. Our results identified a post‐translational regulatory mechanism for FOXA1 consistent with the inverse correlation seen in PCa progression. Targeting FOXA1 through SKP2 inhibition (SZL P1‐41) can restore FOXA1 levels and luminal positive phenotypes enhancing therapeutic sensitivity in advanced stages of PCa.

## Conflict of interest

The authors declare no conflict of interest.

## Author contributions

Conception and design: Z Chen. Acquisition of data: SIC, GL, LJC, WL, TK, WF, Z Cao, NS, NM, LKB, ZAM, and MGI. Analysis and interpretation of data: SIC, GL, LJC, MGI, BRB, XZ, SEA, RJM, XW, and Z Chen. Writing, review, and/or revision of the manuscript: SIC, LJC, and Z Chen.

## Supporting information


**Fig. S1.** SKP2 and FOXA1 colocalize in normal and prostate cancer tissue.
**Fig. S2.** Reverse correlation between SKP2 and FOXA1 in human PCa TMA.
**Fig. S3.** SKP2 is elevated in prostate adenocarcinoma.
**Fig. S4.** SKP2 KD in C4‐2B and 22Rv1 PCa cells.
**Fig. S5.** SKP2 promotes K6‐ and K29‐linked ubiquitination.
**Fig. S6.** SKP2 overexpression increases FOXA1 ubiquitination.
**Fig. S7.** SKP2 overexpression decreases FOXA1 protein by increasing FOXA1 ubiquitination.
**Fig. S8.** SKP2 KD decreases FOXA1 ubiquitination by increasing FOXA1 protein stability.
**Fig. S9.** Prostate tumors of *Pten* ^*pc−/−*^; *Trp53* ^*pc−/−*^; *Skp2* ^*−/−*^ mice have increased Foxa1 protein levels.
**Fig. S10.** SKP2 inhibition decreases FOXA1 ubiquitination.
**Fig. S11.** Ubiquitination of FOXA1 decreases after SKP2 KD and inhibition.
**Fig. S12.** Colocalization of SKP2, FOXA1, and PCNA decreases after SKP2 inhibition in 22Rv1 xenograft mice.
**Fig. S13.** FOXA1 ubiquitination by SKP2 occurs in the C‐terminal TAD.
**Fig. S14.** Ubpred predicted ubiquitination sites for FOXA1.
**Fig. S15.** The effects of FOXA1 mutation on ubiquitination.
**Fig. S16.** FOXA1 protein levels increase upon lysosomal inhibition.
**Fig. S17.** Lysosomal inhibition abrogates effects of SKP2 overexpression on FOXA1.
**Fig. S18.** SKP2, FOXA1, and LAMP2 colocalize in 22Rv1 cells.
**Fig. S19.** *Skp2* mRNA levels decrease in *Pten/Trp53/Skp2* triple‐null MEFs.
**Fig. S20.** Protein stability for Foxa1 increases in *Pten/Trp53/Skp2* triple‐null MEFs.
**Table S1.** Genotyping PCR primer sequences.
**Table S2.** Real‐time quantitative PCR and shRNA primer sequences.

## Data Availability

The data generated for this study are included in this published article and its additional files (supplementary information). All data are available and will be collaboratively shared upon reasonable request.
