# Supplementary material for: Lysosome‐dependent FOXA1 ubiquitination contributes to luminal lineage of advanced prostate cancer
Source: Mol Oncol. 2023 Aug 21;17(10):2126–46. doi: 10.1002/1878-0261.13497 (PMC10552895; doi:10.1002/1878-0261.13497)
Supplement: Supplementary file 1 — Fig. S1. SKP2 and FOXA1 colocalize in normal and prostate cancer tissue. Fig. S2. Reverse correlation between SKP2 and FOXA1 in human PCa TMA. Fig. S3. SKP2 is elevated in prostate adenocarcinoma. Fig. S4. SKP2 KD in C4‐2B and 22Rv1 PCa cells. Fig. S5. SKP2 promotes K6‐ and K29‐linked ubiquitination. Fig. S6. SKP2 overexpression increases FOXA1 ubiquitination. Fig. S7. SKP2 overexpression decreases FOXA1 protein by increasing FOXA1 ubiquitination. Fig. S8. SKP2 KD decreases FOXA1 ubiquitination by increasing FOXA1 protein stability. Fig. S9. Prostate tumors of Pten pc−/− ; Trp53 pc−/− ; Skp2 −/− mice have increased Foxa1 protein levels. Fig. S10. SKP2 inhibition decreases FOXA1 ubiquitination. Fig. S11. Ubiquitination of FOXA1 decreases after SKP2 KD and inhibition. Fig. S12. Colocalization of SKP2, FOXA1, and PCNA decreases after SKP2 inhibition in 22Rv1 xenograft mice. Fig. S13. FOXA1 ubiquitination by SKP2 occurs in the C‐terminal TAD. Fig. S14. Ubpred predicted ubiquitination sites for FOXA1. Fig. S15. The effects of FOXA1 mutation on ubiquitination. Fig. S16. FOXA1 protein levels increase upon lysosomal inhibition. Fig. S17. Lysosomal inhibition abrogates effects of SKP2 overexpression on FOXA1. Fig. S18. SKP2, FOXA1, and LAMP2 colocalize in 22Rv1 cells. Fig. S19. Skp2 mRNA levels decrease in Pten/Trp53/Skp2 triple‐null MEFs. Fig. S20. Protein stability for Foxa1 increases in Pten/Trp53/Skp2 triple‐null MEFs. Table S1. Genotyping PCR primer sequences. Table S2. Real‐time quantitative PCR and shRNA primer sequences. [file MOL2-17-2126-s001.zip › _MOLONC-22-0816-CELADA_SUPPLEMENTARY_Figures.pdf]

Supplementary Figure S1

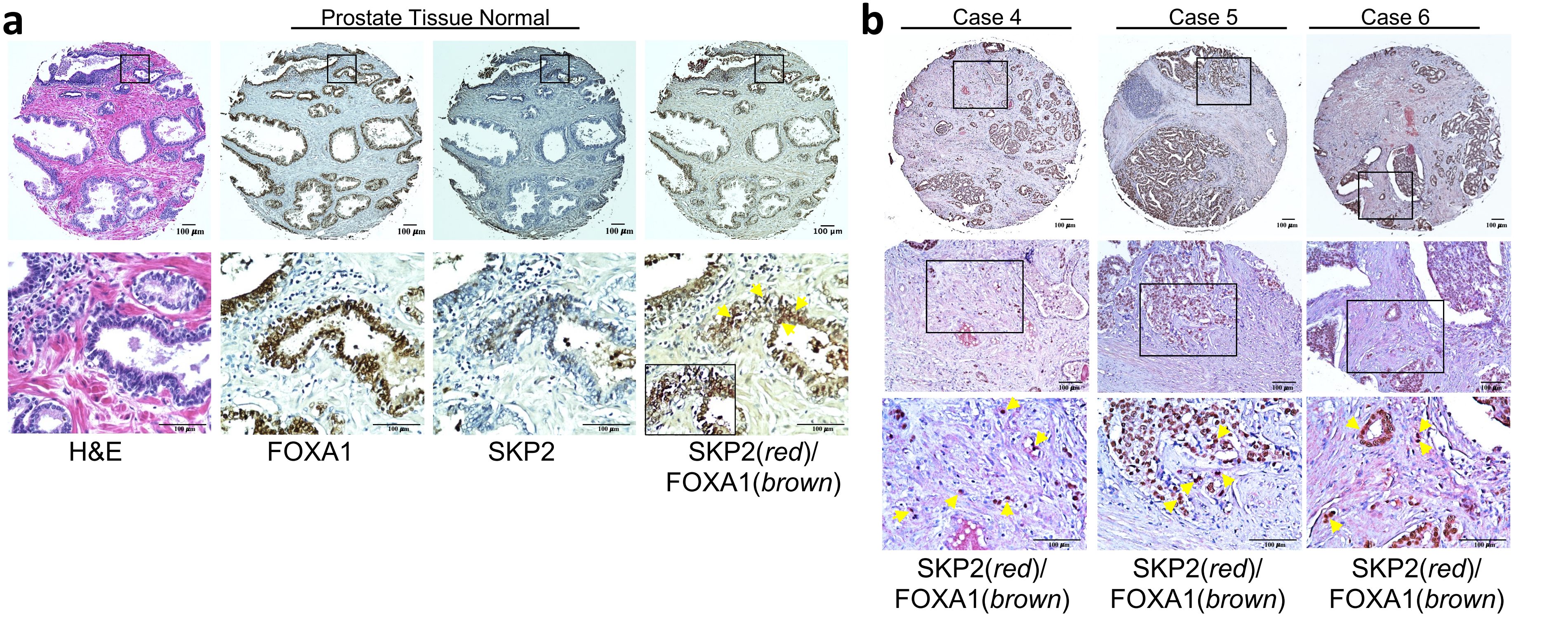

Supplementary Figure S2

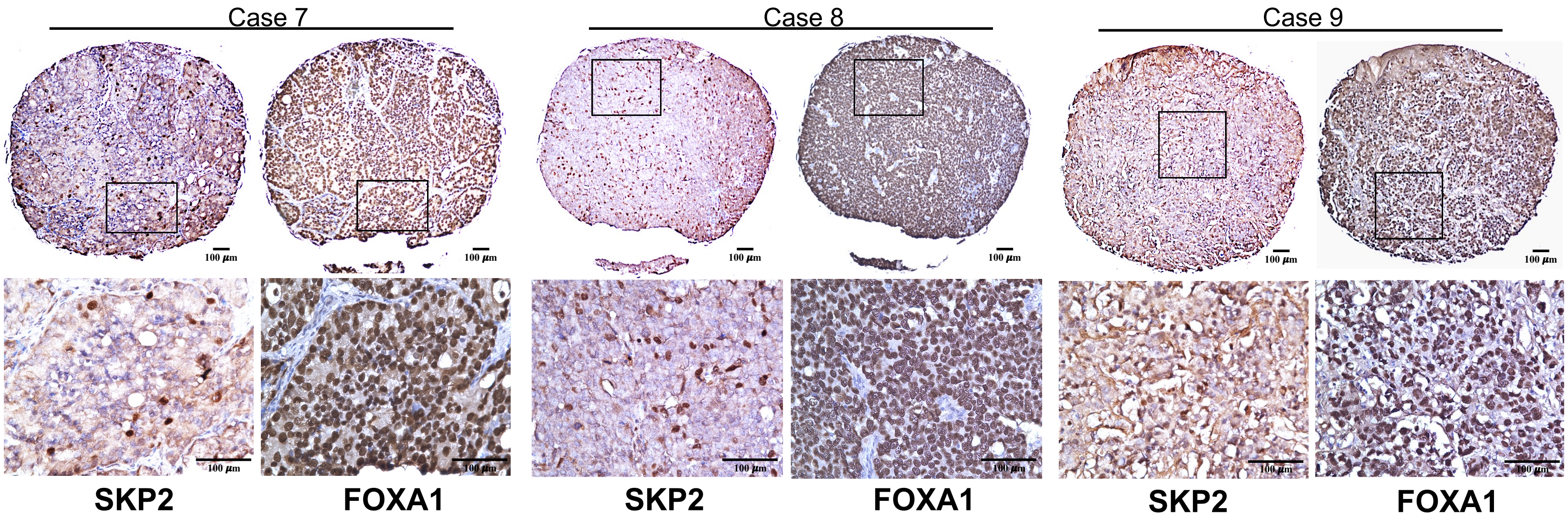

FOXA1 detection level (Intensity score)	SKP2 detection level (Intensity score)			
	0	1	2	3
0	9	0	0	0
1	1	0	3	2
2	4	5	2	2
3	22	30	0	0
Degrees of freedom: 9; Chi: 56.16; P-value:<0.0001				

Supplementary Figure S3

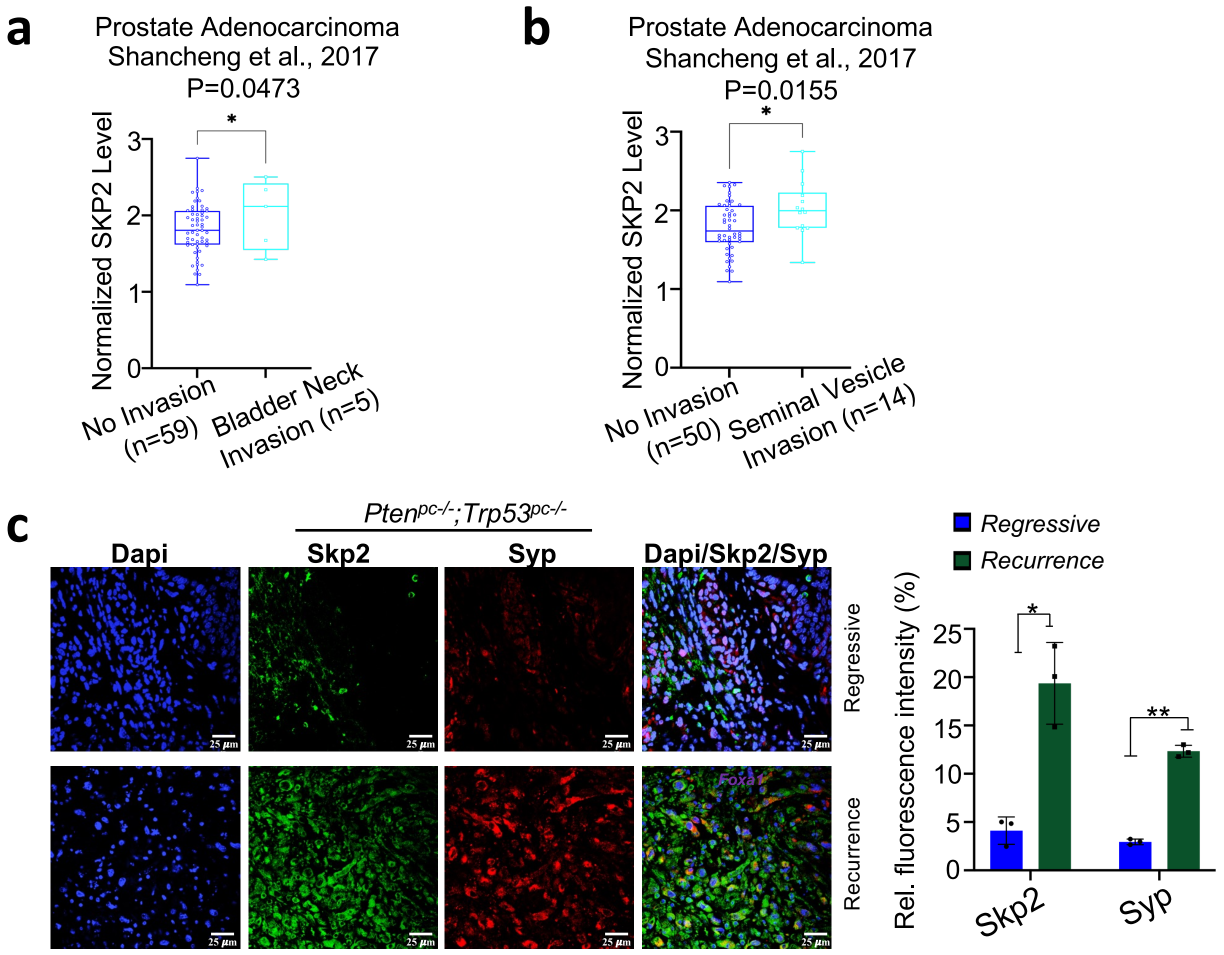

Supplementary Figure S4

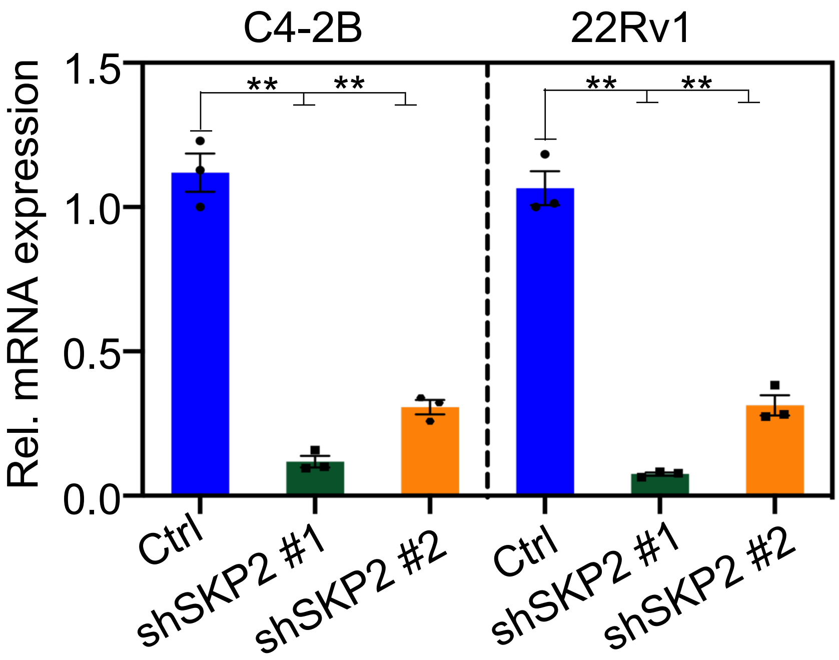

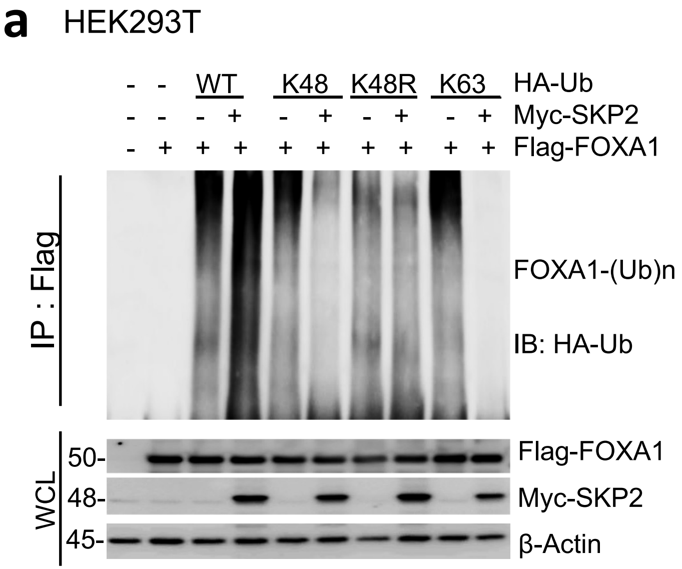
a
HEK293T
- - WT K48 K48R K63 HA-Ub
- - - + - + - + - + Myc-SKP2
- + + + + + + + + + Flag-FOXA1
IP : Flag
FOXA1-(Ub)n
IB: HA-Ub
WCL
50-
48-
45-
Flag-FOXA1
Myc-SKP2
β-Actin

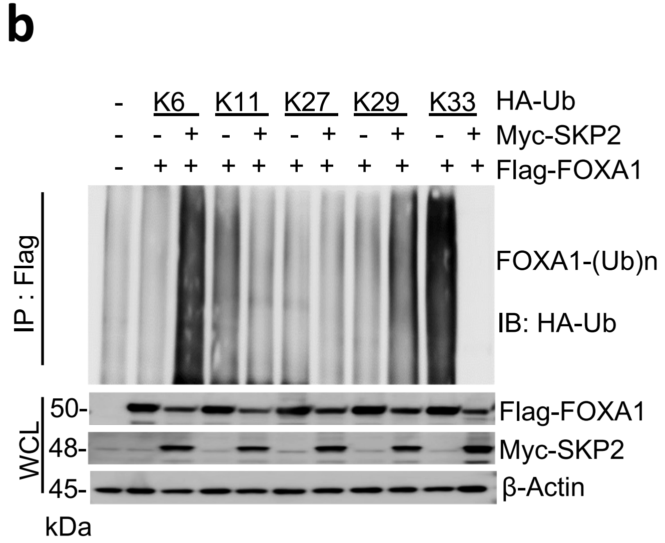
b
- K6 K11 K27 K29 K33 HA-Ub
- - + - + - + - + - + Myc-SKP2
- + + + + + + + + + + Flag-FOXA1
IP : Flag
FOXA1-(Ub)n
IB: HA-Ub
WCL
50-
48-
45-
Flag-FOXA1
Myc-SKP2
β-Actin
kDa

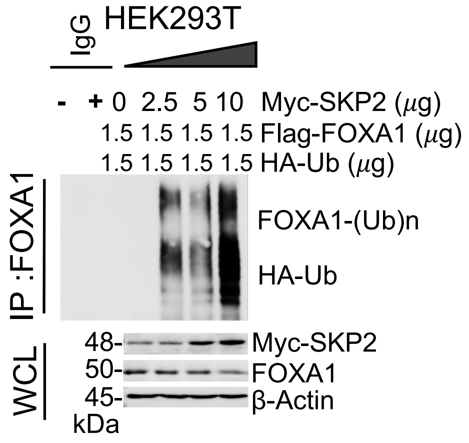

HEK293T
IgG
- + 0 2.5 5 10 Myc-SKP2 (μg)
1.5 1.5 1.5 1.5 Flag-FOXA1 (μg)
1.5 1.5 1.5 1.5 HA-Ub (μg)
IP :FOXA1
FOXA1-(Ub)n
HA-Ub
WCL
48- Myc-SKP2
50- FOXA1
45- β-Actin
kDa

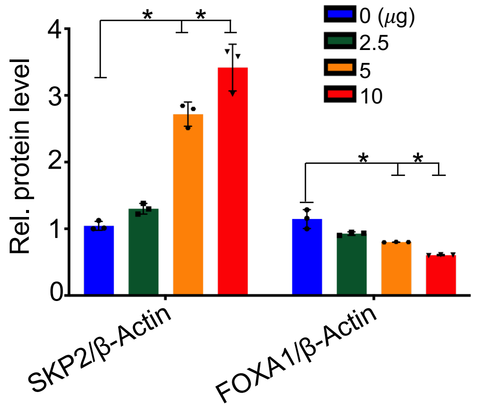

Rel. protein level
0
1
2
3
4
*
*
*
*
0 (μg)
2.5
5
10
SKP2/β-Actin
FOXA1/β-Actin

Supplementary Figure S7

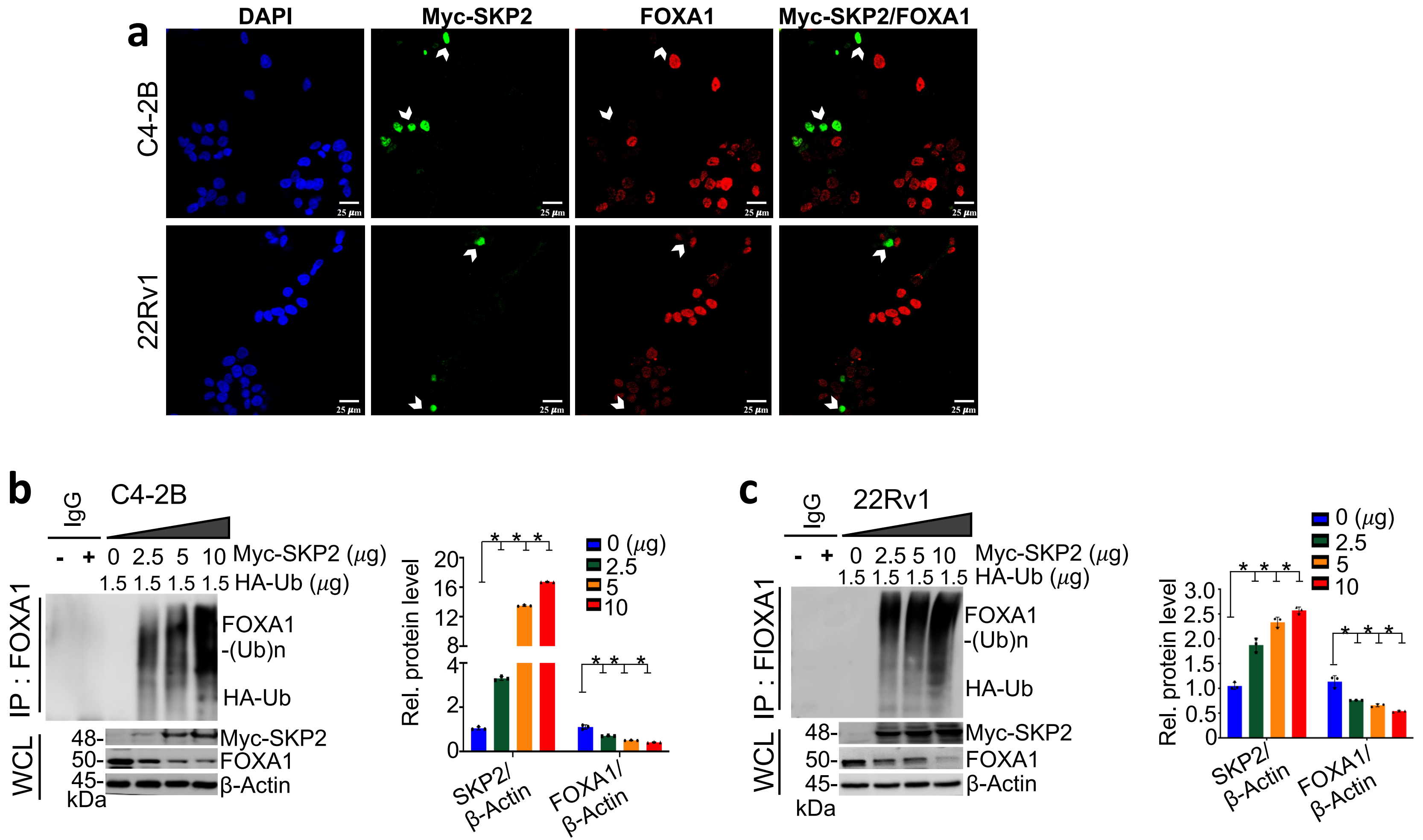

Supplementary Figure S8

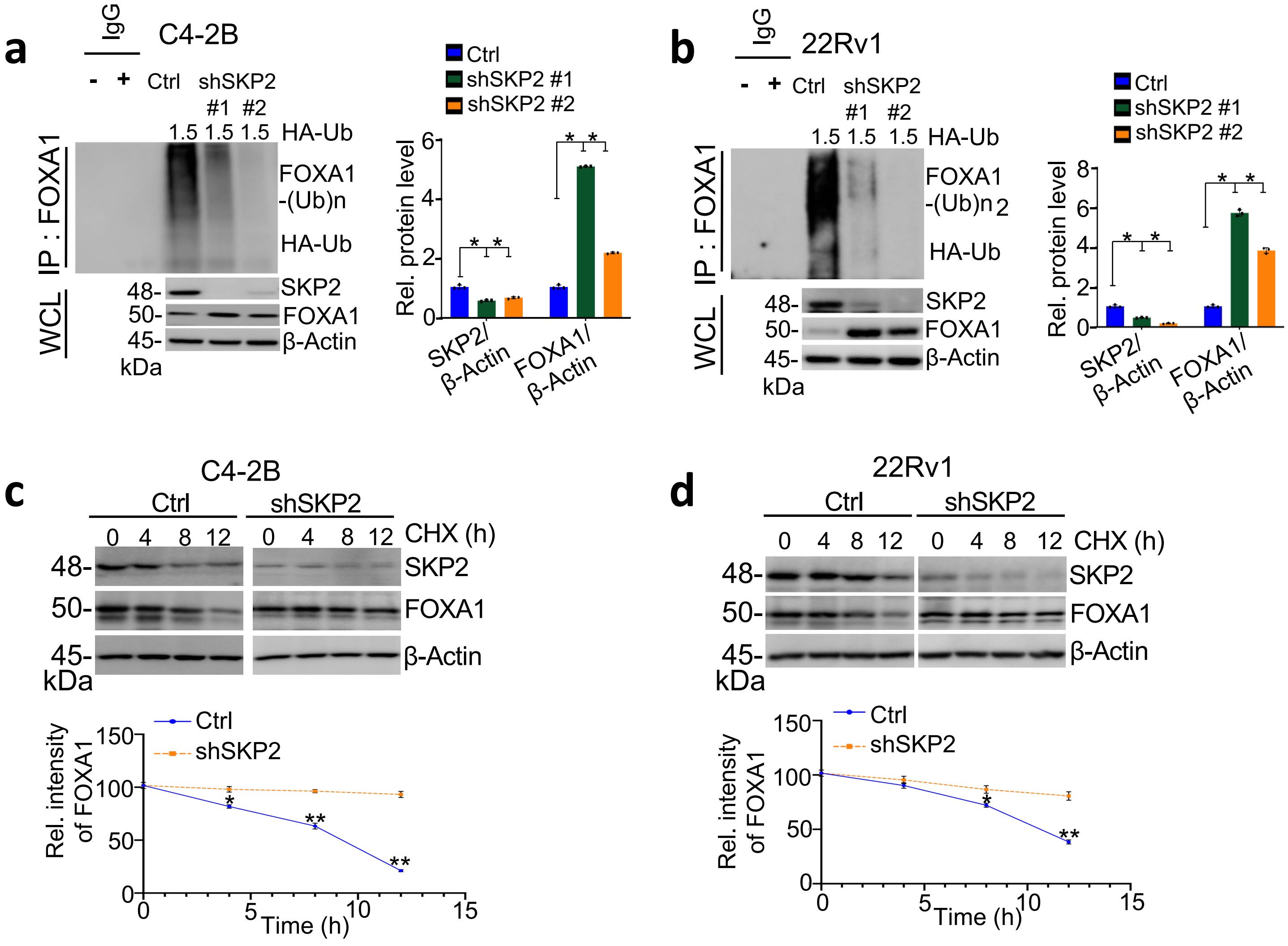

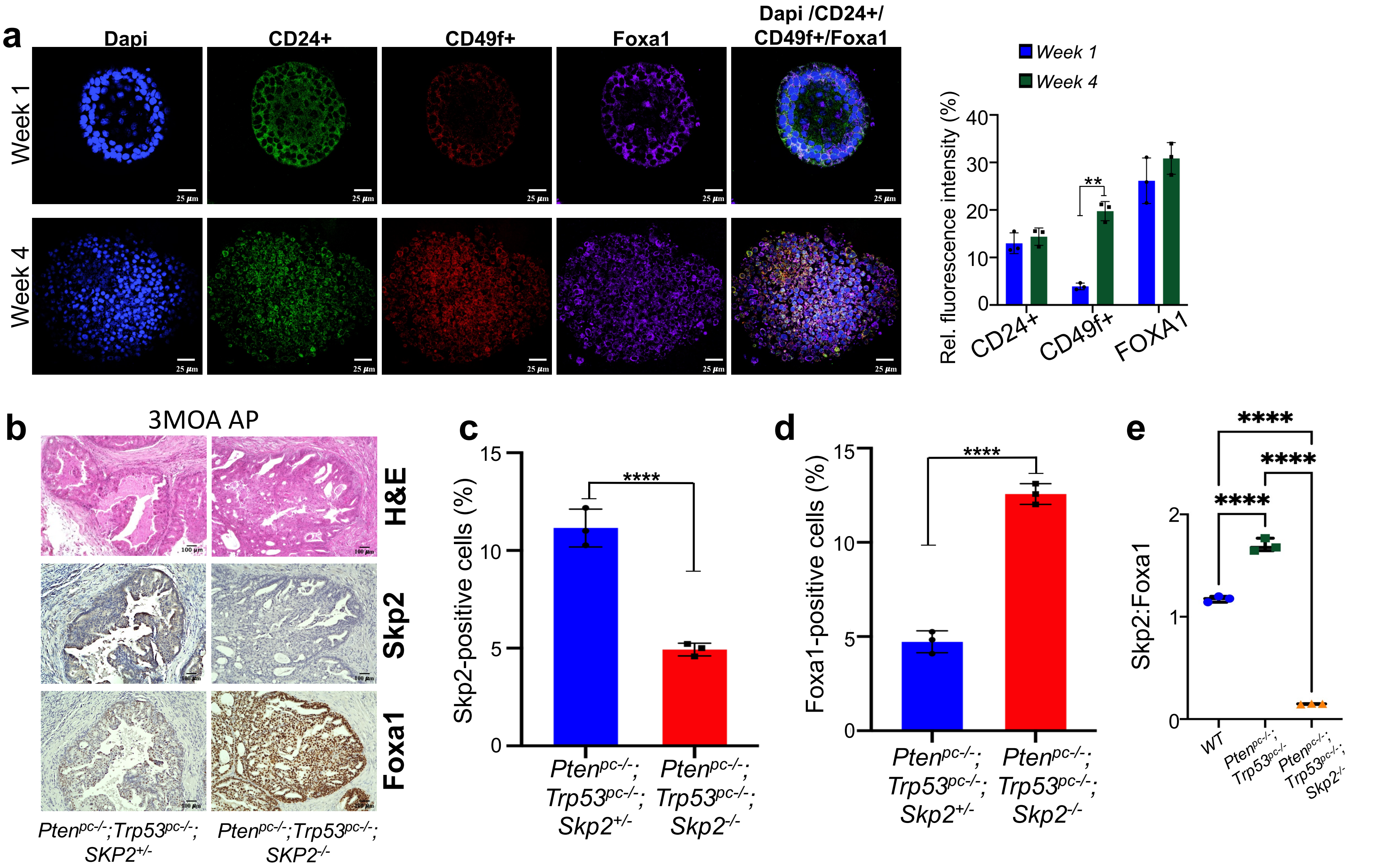
a
Dapi
CD24+
CD49f+
Foxa1
Dapi /CD24+/
CD49f+/Foxa1
Week 1
Week 4
25 μm
Week 1
Week 4
Rel. fluorescence intensity (%)
40
30
20
10
0
**
CD24+
CD49f+
FOXA1
b
3MOA AP
H&E
Skp2
Foxa1
Ptenpc-/-;Trp53pc-/-; SKP2+/-
Ptenpc-/-;Trp53pc-/-; SKP2-/-
c
Skp2-positive cells (%)
15
10
5
0
****
Ptenpc-/-; Trp53pc-/-; Skp2+/-
Ptenpc-/-; Trp53pc-/-; Skp2-/-
d
Foxa1-positive cells (%)
15
10
5
0
****
Ptenpc-/-; Trp53pc-/-; Skp2+/-
Ptenpc-/-; Trp53pc-/-; Skp2-/-
e
Skp2:Foxa1
2
1
0
****
****
****
WT
Ptenpc-/-; Trp53pc-/-
Ptenpc-/-; Trp53pc-/-; Skp2-/-

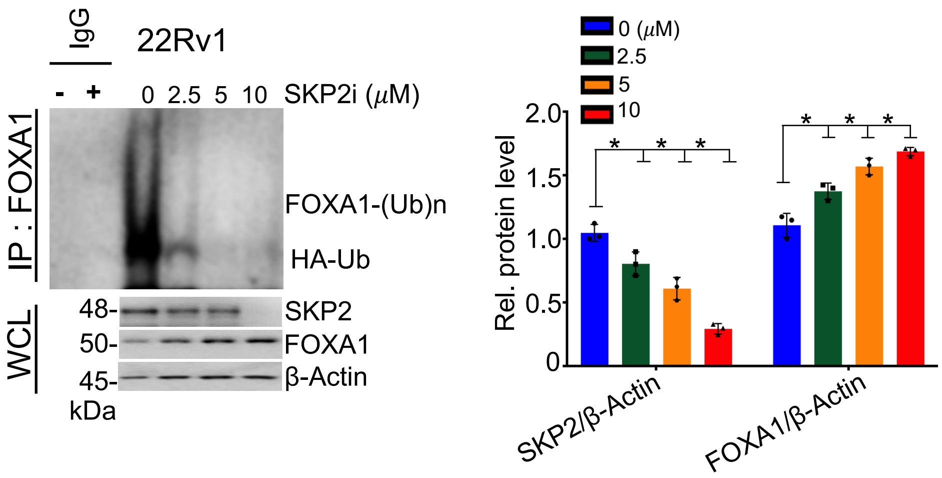
IgG
22Rv1
- + 0 2.5 5 10 SKP2i (μM)
IP : FOXA1
FOXA1-(Ub)n
HA-Ub
WCL
48- SKP2
50- FOXA1
45- β-Actin
kDa
0 (μM)
2.5
5
10
Rel. protein level
2.0
1.5
1.0
0.5
0
*
SKP2/β-Actin
FOXA1/β-Actin

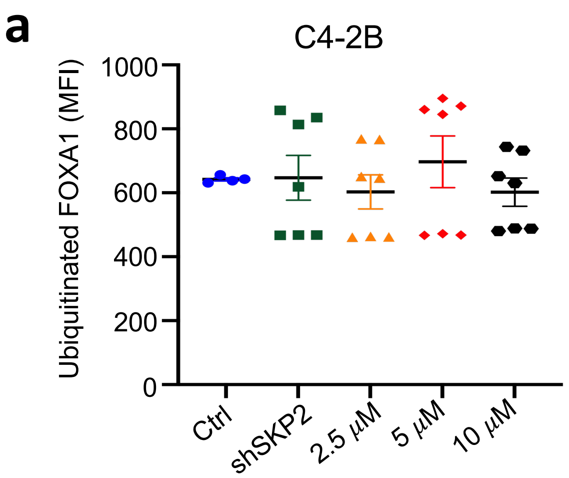
a
C4-2B
Ubiquitinated FOXA1 (MFI)
1000
800
600
400
200
0
Ctrl
shSKP2
2.5 μM
5 μM
10 μM

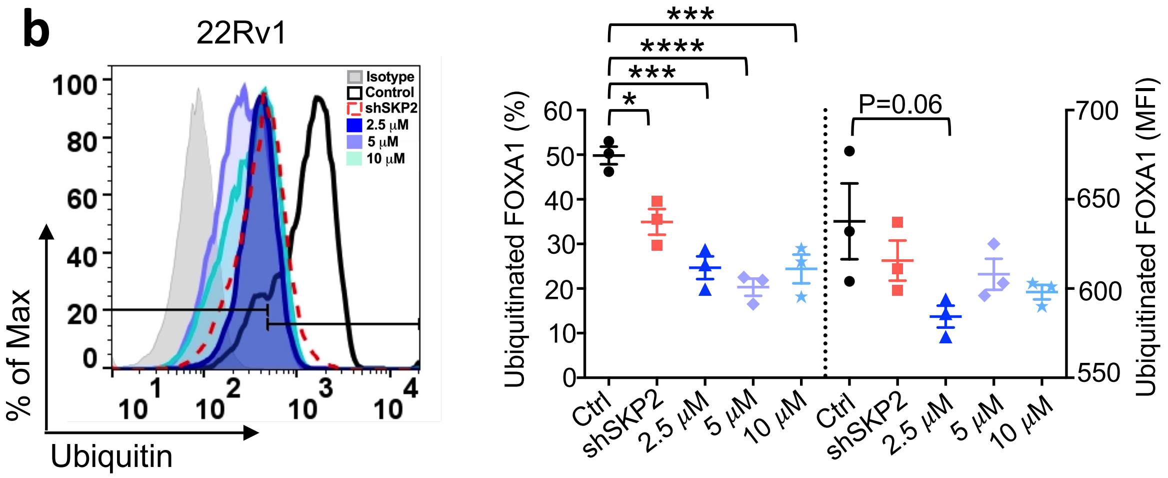
b
22Rv1
Isotype
Control
shSKP2
2.5 μM
5 μM
10 μM
% of Max
Ubiquitin
***
****
***
*
P=0.06
Ubiquitinated FOXA1 (%)
Ubiquitinated FOXA1 (MFI)
Ctrl
shSKP2
2.5 μM
5 μM
10 μM

Supplementary Figure S12

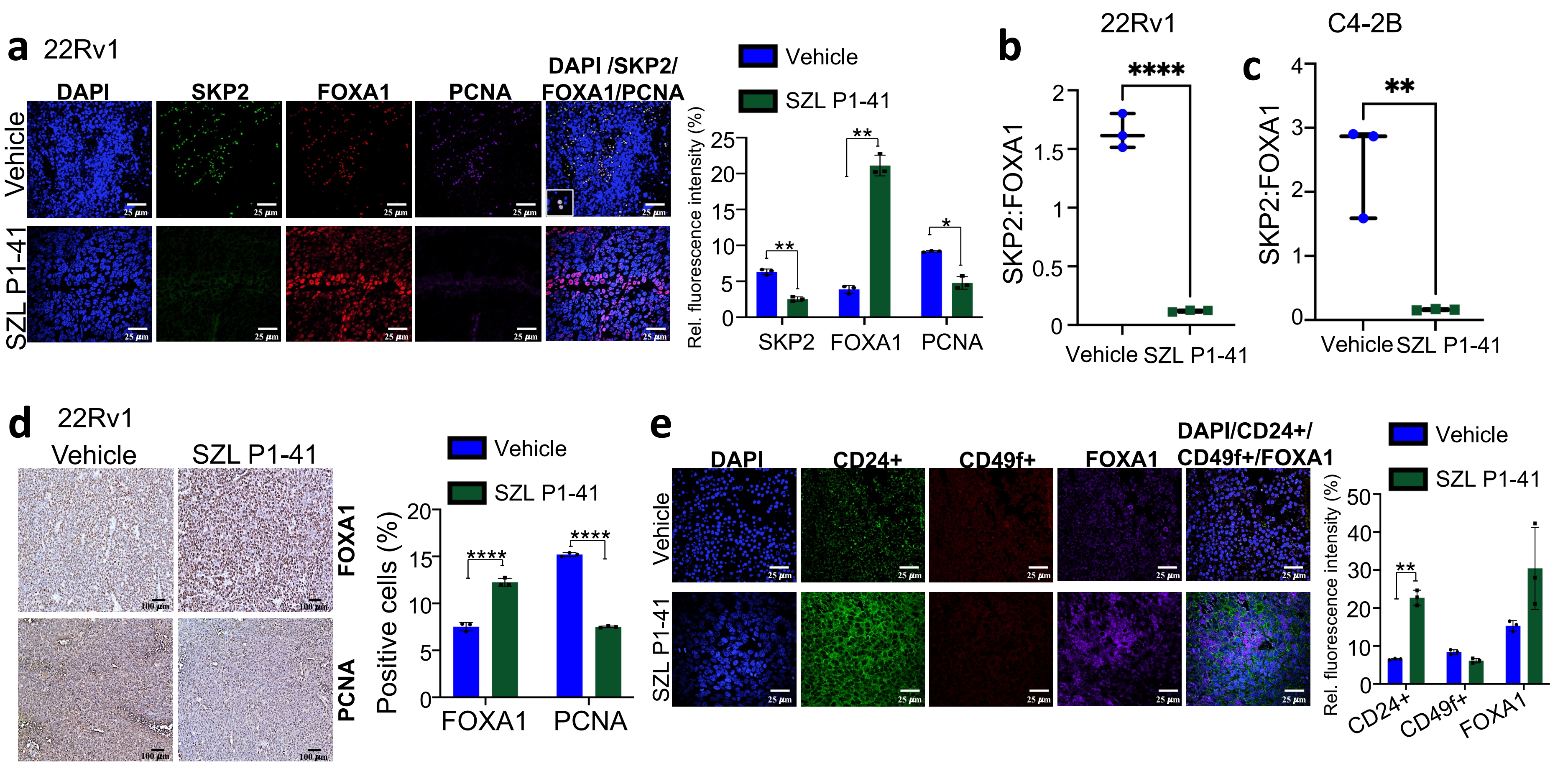

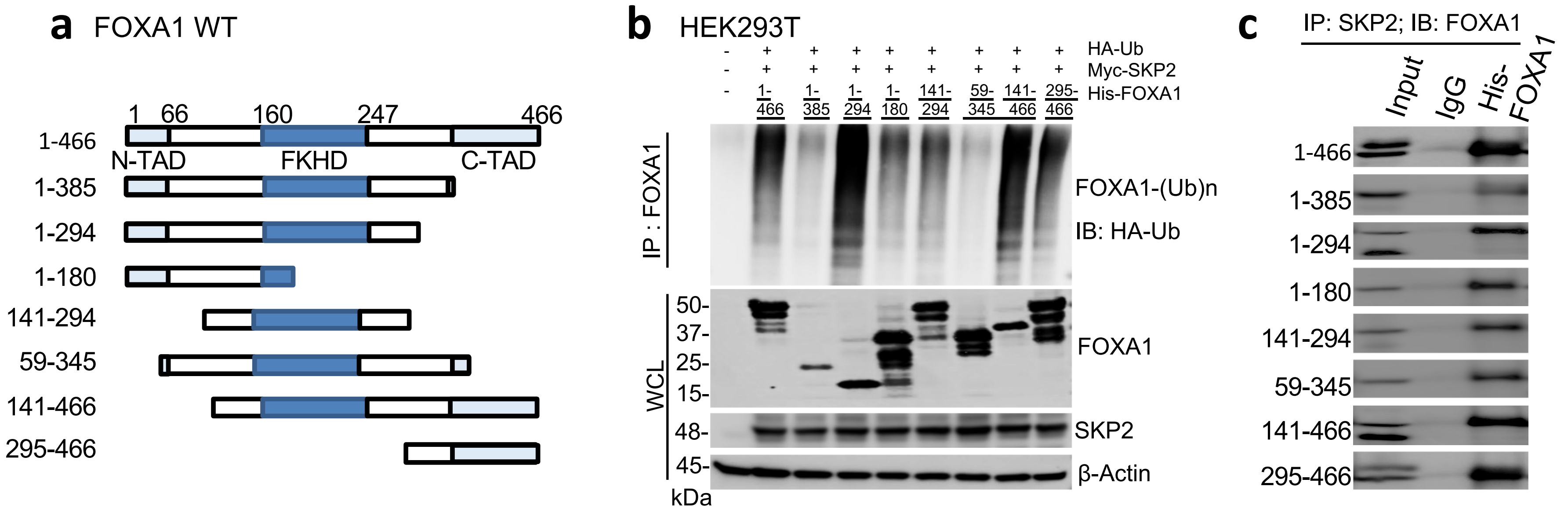
a FOXA1 WT
1 66 160 247 466
1-466
N-TAD FKHD C-TAD
1-385
1-294
1-180
141-294
59-345
141-466
295-466
b HEK293T
HA-Ub
Myc-SKP2
His-FOXA1
1-466 1-385 1-294 1-180 141-294 59-345 141-466 295-466
IP : FOXA1
FOXA1-(Ub)n
IB: HA-Ub
WCL
50- 37- 25- 15-
FOXA1
48-
SKP2
45-
β-Actin
kDa
c IP: SKP2; IB: FOXA1
Input IgG His-FOXA1
1-466
1-385
1-294
1-180
141-294
59-345
141-466
295-466

Residue	Score	Ubiquitinated
414 ☆	0.84	High Confidence
316	0.81	Medium Confidence
350	0.78	Medium Confidence
295	0.77	Medium Confidence
418	0.76	Medium Confidence
288	0.73	Medium Confidence
6	0.69	Medium Confidence
389	0.64	Low Confidence

Label	Score range	Sensitivity	Specificity
High confidence	0.84 ≤ s ≤ 1.00	0.197	0.989
Medium confidence	0.69 ≤ s ≤ 0.84	0.346	0.950
Low confidence	0.62 ≤ s ≤ 0.69	0.464	0.903

UbPred: prediction for ubiquitination sites (http://www.ubpred.org/)

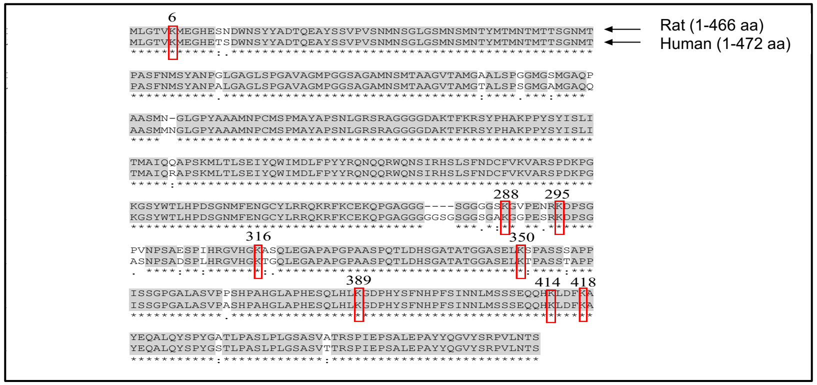

NCBI Blast:

Identities: 444/473 (94%)
Positives: 455/473 (96%)
Gaps: 5/473 (1%)
E value : 0.0
Percent identical: 93.87%
Score: 793 bits (2047)

ClustalW:

Percent Identical: 98.8034%
Alignment score: 2784

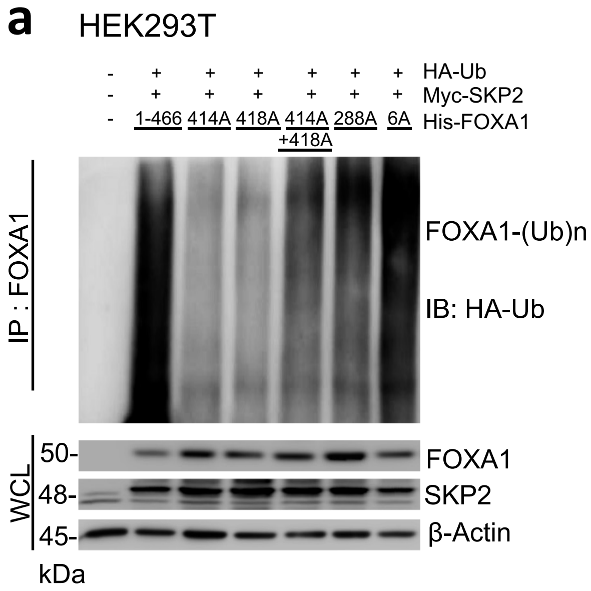
a
HEK293T
HA-Ub
Myc-SKP2
His-FOXA1
1-466
414A
418A
414A +418A
288A
6A
IP : FOXA1
FOXA1-(Ub)n
IB: HA-Ub
WCL
50-
48-
45-
FOXA1
SKP2
β-Actin
kDa

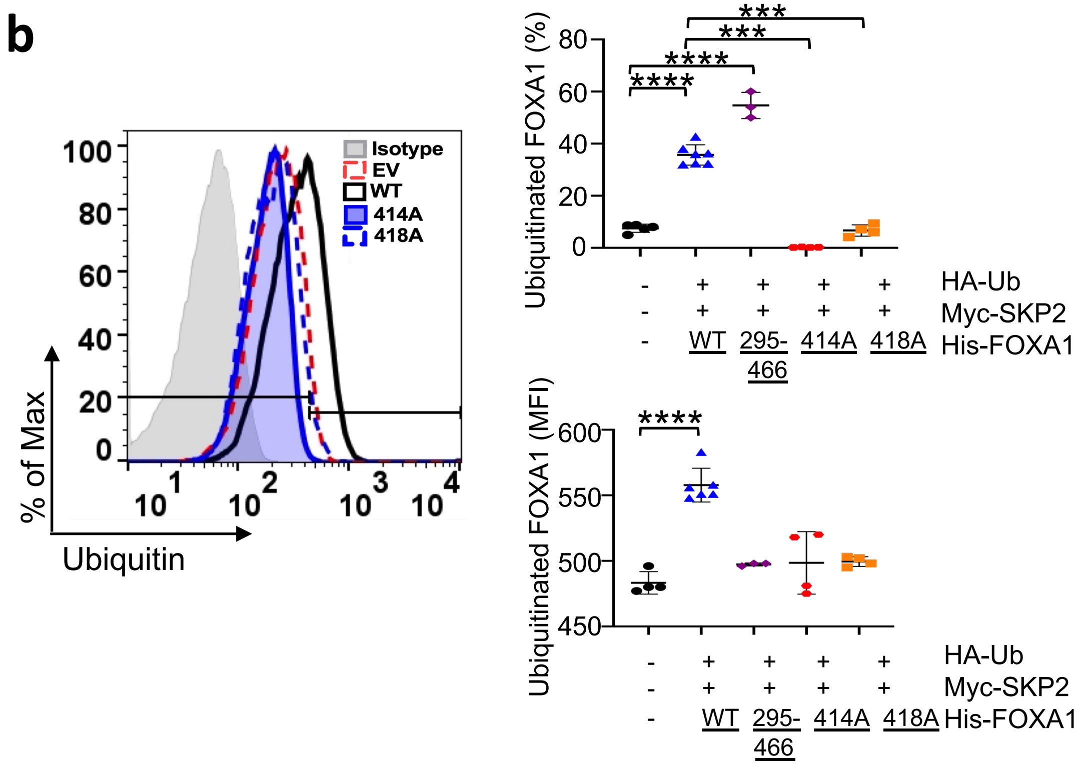
b
Isotype
EV
WT
414A
418A
% of Max
Ubiquitin
Ubiquitinated FOXA1 (%)
****
***
HA-Ub
Myc-SKP2
His-FOXA1
WT
295-466
414A
418A
Ubiquitinated FOXA1 (MFI)

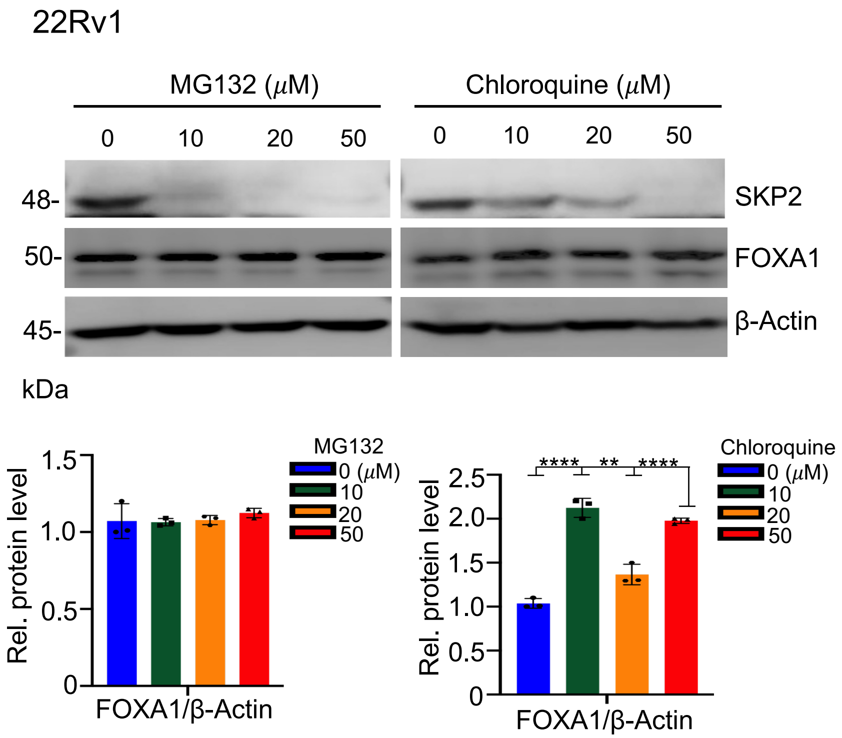
22Rv1
MG132 (μM)
Chloroquine (μM)
0
10
20
50
0
10
20
50
48-
50-
45-
kDa
SKP2
FOXA1
β-Actin
MG132
0 (μM)
10
20
50
Rel. protein level
1.5
1.0
0.5
0
FOXA1/β-Actin
Chloroquine
0 (μM)
10
20
50
****
**
****
Rel. protein level
2.5
2.0
1.5
1.0
0.5
0
FOXA1/β-Actin

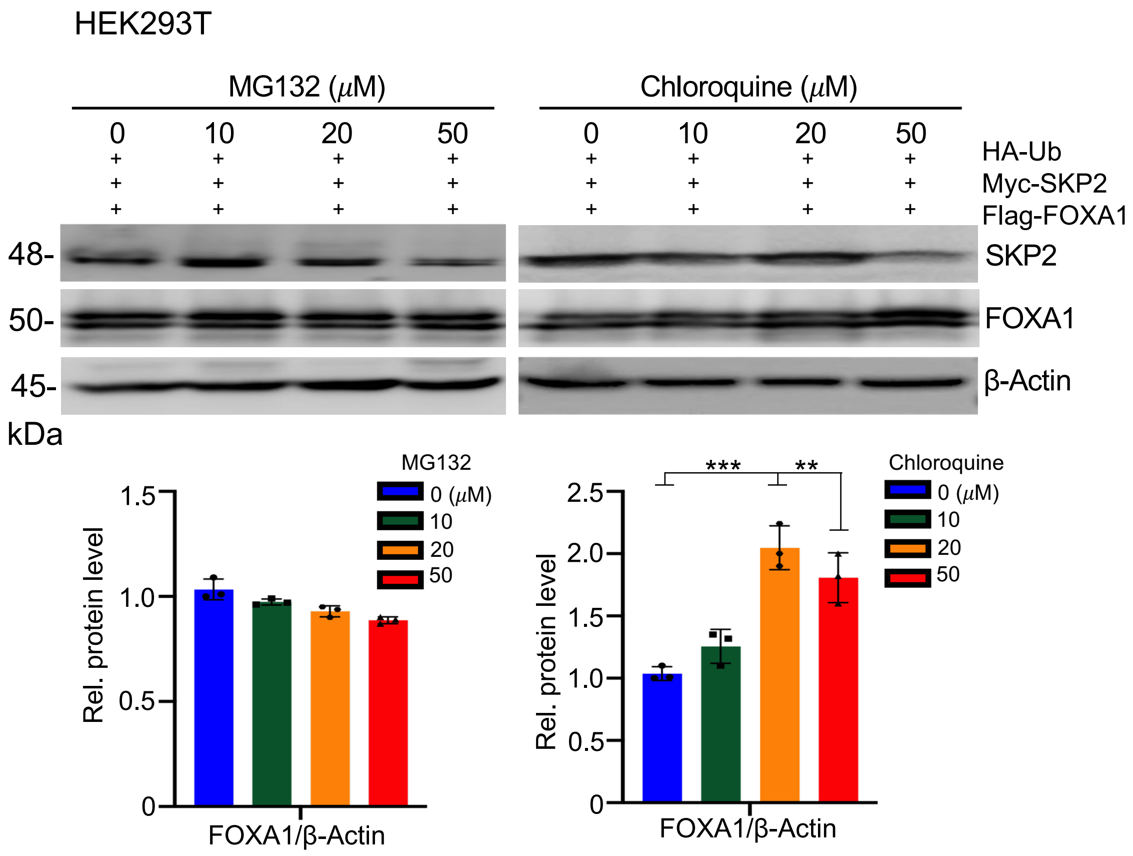
HEK293T
MG132 (μM)
Chloroquine (μM)
0
10
20
50
HA-Ub
Myc-SKP2
Flag-FOXA1
48-
50-
45-
kDa
SKP2
FOXA1
β-Actin
MG132
0 (μM)
10
20
50
Rel. protein level
1.5
1.0
0.5
0
FOXA1/β-Actin
Chloroquine
***
**
2.5
2.0
1.5
1.0
0.5
0

Supplementary Figure S18

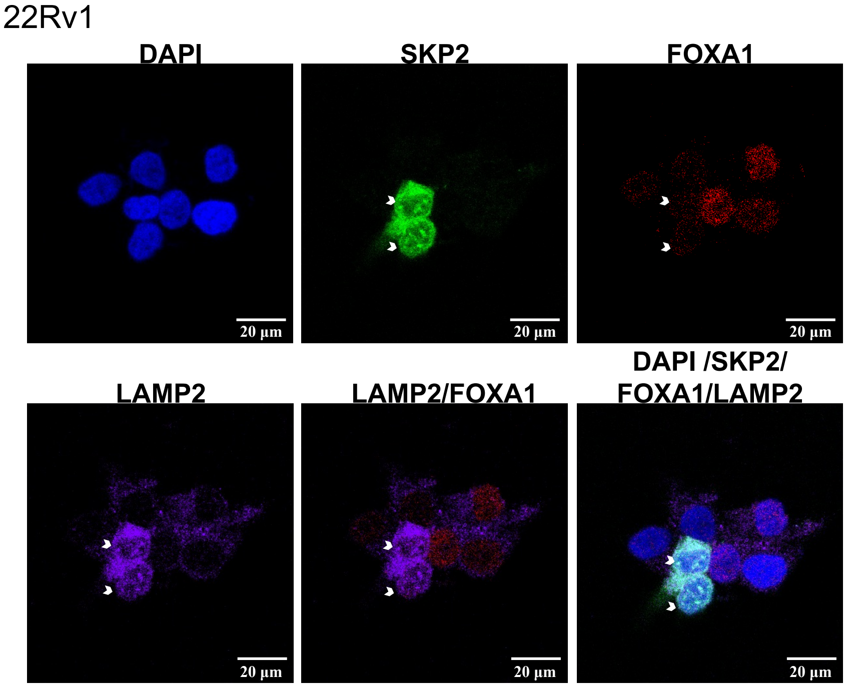

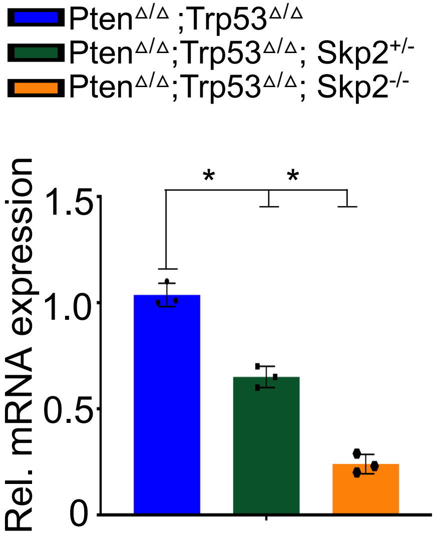
Pten$^{\Delta/\Delta}$;Trp53$^{\Delta/\Delta}$
Pten$^{\Delta/\Delta}$;Trp53$^{\Delta/\Delta}$; Skp2$^{+/-}$
Pten$^{\Delta/\Delta}$;Trp53$^{\Delta/\Delta}$; Skp2$^{-/-}$
Rel. mRNA expression
1.5
1.0
0.5
0
*
*

Supplementary Figure S20

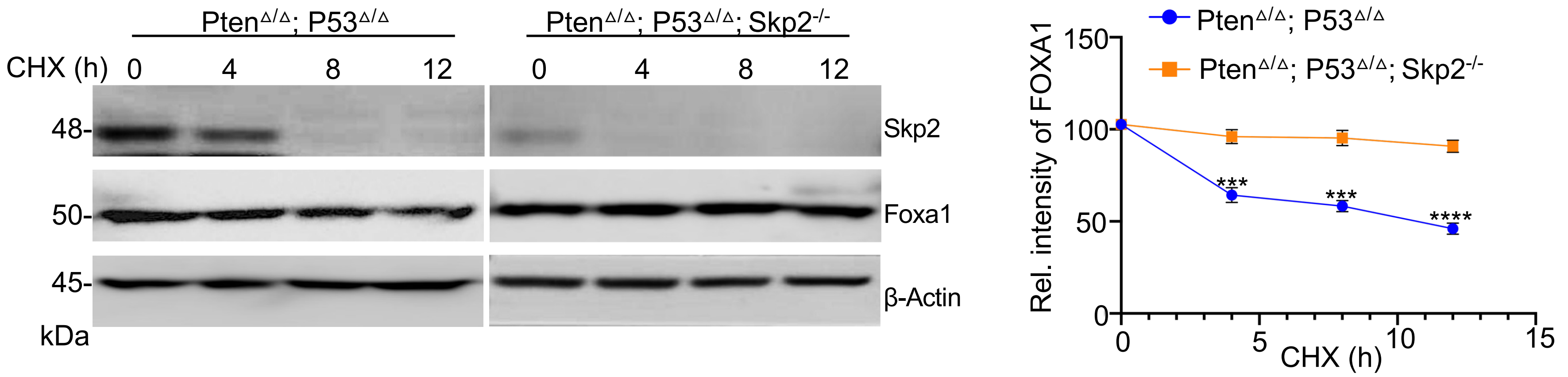

Supplementary Table S1: Genotyping PCR primer sequences

Primer Name	Primer Sequence	Ref.
Pten-forward	5’ TGTTTTTGACCAATTAAAGTAGGCTGTG 3’	[5]
Pten-reverse	5’ AAAAGTTCCCCTGCTGATGATTTGT 3’	[5]
Cre-forward	5’ TGATGGACATGTTCAGGGATC 3’	[5]
Cre-reverse	5’ CAGCCACCAGCTTGCATGA 3’	[5]
SKP2-WT-forward	5’ AGAGTGGAAGAACCCAGGCAGGAC 3’	[4]
SKP2-WT-reverse	5’ CCCGTGGAGGGAAAAAGAGGGACG 3’	[4]
SKP2-KO-forward	5’ GCATCGCCTTCTATCGCCTTCTTG 3’	[4]
SKP2-KO-reverse	5’ TTCCCACCCCCACATCCAGTCATT 3’	[4]

Supplementary Table S2:Real-time quantitative PCR and shRNA primer sequences

RT-qPCR Primer Name	Primer Sequence	Ref.
SKP2-mRNA-forward	5’ CCC ACG GAA ACG GCT GAA GA 3’	[1]
SKP2-mRNA-reverse	5’ CGC TAG GCG ATA CCA CCT CTT ACA A 3’	[1]
Actin-mRNA-forward	5’ CTG AAG TAC CCC ATC GAG CAC GGC A 3’	[2]
Actin-mRNA-reverse	5’ GGA TAG CAC AGC CTG GAT AGC AAC G 3’	[2]
Hexb-mRNA-forward	5’ CTG GTG TCG CTA GTG TCG C 3’	PrimerBank ID: 6754186a1
Hexb-mRNA-reverse	5’ CAG GGC CAT GAT GTC TCT TG 3’	PrimerBank ID: 6754186a1
Mcoln1-mRNA-forward	5’ CTG ACC CCC AAT CCT GGG TAT 3’	PrimerBank ID: 16716463a1
Mcoln1-mRNA-reverse	5’ GGC CCG GAA CTT GTC ACA T3’	PrimerBank ID: 16716463a1
**SKP2 shRNA Primer Name**	**Primer Sequence**	**Ref**
SKP2-shRNA1-S	CCGGGCCTAAGCTAAATCGAGAGAACTCGAGTTCTCTCGATTTAGCTTAGGCTTTTTG	[3]
SKP2-shRNA1-AS	AATTCAAAAAGCCTAAGCTAAATCGAGAGAACTCGAGTTCTCTCGATTTAGCTTAGGC	[3]
SKP2-shRNA2-S	CCGGGATAGTGTCATGCTAAAGAATCTCGAGATTCTTTAGCATGACACTATCTTTTTG	[4]
SKP2-shRNA2-AS	AATTCAAAAAGATAGTGTCATGCTAAAGAATCTCGAGATTCTTTAGCATGACACTATC	[4]
SKP2-scramble-S	CCGGATGAGTCAACGCGAATACAGACTCGAGTC TGTATTCGCGTTGACTCATTTTTTTG	
SKP2-scramble-AS	AATTCAAAAAAATGAGTCAACGCGAATACAGACT CGAGTCTGTATTCGCGTTGACTCAT	

Supplementary References

1. Bhatt K, Hu R, Spofford L, Aplin A. Mutant B-RAF signaling and cyclin D1 regulate Cks1/S-phase kinase-associated protein 2-mediated degradation of p27 Kip1 in human melanoma cells. Oncogene. 2007;26(7):1056.
2. Hoppe B, Conti-Tronconi B, Horton R. Gel-loading dyes compatible with PCR. Biotechniques. 1992;12(5):679-80.
3. Wu J, Lee S-W, Zhang X, Han F, Kwan S-Y, Yuan X, et al. Foxo3a transcription factor is a negative regulator of Skp2 and Skp2 SCF complex. Oncogene. 2013;32(1):78.
4. Lin H-K, Chen Z, Wang G, Nardella C, Lee S-W, Chan C-H, et al. Skp2 targeting suppresses tumorigenesis by Arf-p53-independent cellular senescence. Nature. 2010;464(7287):374.
5. Chen Z, Trotman LC, Shaffer D, Lin HK, Dotan ZA, Niki M, Koutcher JA, Scher HI, Ludwig T, Gerald W, Cordon-Cardo C, Pandolfi PP. Crucial role of p53-depen- dent cellular senescence in suppression of Pten-deficient tumorigenesis. Nature. 2005; 436:725–730.
