# Supplementary material for: Lysosome‐dependent FOXA1 ubiquitination contributes to luminal lineage of advanced prostate cancer
Source: Mol Oncol. 2023 Aug 21;17(10):2126–46. doi: 10.1002/1878-0261.13497 (PMC10552895; doi:10.1002/1878-0261.13497)
Supplement: Supplementary file 1 — Fig. S1. SKP2 and FOXA1 colocalize in normal and prostate cancer tissue. Fig. S2. Reverse correlation between SKP2 and FOXA1 in human PCa TMA. Fig. S3. SKP2 is elevated in prostate adenocarcinoma. Fig. S4. SKP2 KD in C4‐2B and 22Rv1 PCa cells. Fig. S5. SKP2 promotes K6‐ and K29‐linked ubiquitination. Fig. S6. SKP2 overexpression increases FOXA1 ubiquitination. Fig. S7. SKP2 overexpression decreases FOXA1 protein by increasing FOXA1 ubiquitination. Fig. S8. SKP2 KD decreases FOXA1 ubiquitination by increasing FOXA1 protein stability. Fig. S9. Prostate tumors of Pten pc−/− ; Trp53 pc−/− ; Skp2 −/− mice have increased Foxa1 protein levels. Fig. S10. SKP2 inhibition decreases FOXA1 ubiquitination. Fig. S11. Ubiquitination of FOXA1 decreases after SKP2 KD and inhibition. Fig. S12. Colocalization of SKP2, FOXA1, and PCNA decreases after SKP2 inhibition in 22Rv1 xenograft mice. Fig. S13. FOXA1 ubiquitination by SKP2 occurs in the C‐terminal TAD. Fig. S14. Ubpred predicted ubiquitination sites for FOXA1. Fig. S15. The effects of FOXA1 mutation on ubiquitination. Fig. S16. FOXA1 protein levels increase upon lysosomal inhibition. Fig. S17. Lysosomal inhibition abrogates effects of SKP2 overexpression on FOXA1. Fig. S18. SKP2, FOXA1, and LAMP2 colocalize in 22Rv1 cells. Fig. S19. Skp2 mRNA levels decrease in Pten/Trp53/Skp2 triple‐null MEFs. Fig. S20. Protein stability for Foxa1 increases in Pten/Trp53/Skp2 triple‐null MEFs. Table S1. Genotyping PCR primer sequences. Table S2. Real‐time quantitative PCR and shRNA primer sequences. [file MOL2-17-2126-s001.zip › MOLONC-22-0816_Supporting Information_Full_legends.pdf]

Supporting Information

Supplementary Figure S1. SKP2 and FOXA1 colocalize in normal and prostate cancer tissue. (a-b) Immunohistochemistry **(**IHC) co-staining for SKP2 (*red*) and FOXA1 (*brown*) protein levels in tissue microarray (TMA) of primary prostate tumors (n=35). Yellow arrows indicate colocalization between SKP2 and FOXA1 protein. Scale bars are 100 μm.

Supplementary Figure S2. Reverse correlation between SKP2 and FOXA1 in human PCa TMA. Immunohistochemistry (IHC) staining for SKP2 and FOXA1 protein levels in hormone refractory PCa TMA (n=80). Scale bars are 100 μm. The corresponding statistical analysis for the Pearson correlation coefficient and Chi-square test between SKP2 and FOXA1 levels can be found below. Protein expression levels for SKP2 and FOXA1 were graded with intensity score 0 (negative stain), 1 (weak stain), 2 (moderate stain) and 3 (strong stain) as described by Lakshmikanthan V, Zou L, Kim JI, Michal A, Nie Z, Messias NC, Benovic JL, Daaka Y. Identification of βArrestin2 as a corepressor of androgen receptor signaling in prostate cancer. Proceedings of the National Academy of Sciences. 2009 Jun 9;106(23):9379-84. Comparison between groups was performed using Student's *t*-test. Bars indicate SEM. * $P < 0.05$, ** $P < 0.01$, *** $P < 0.001$, **** $P < 0.0001$.

Supplementary Figure S3. SKP2 is elevated in prostate adenocarcinoma. (a-b) SKP2 mRNA expression levels in prostate adenocarcinoma bladder neck and seminal vesicle invasion retrieved from cBioPortal (cbioportal.org). The corresponding normalized SKP2 levels were plotted. **(c)** Castrated $Pten^{pc-/-}$; $Trp53^{pc-/-}$ mice undergoing regression and recurrence with increased Skp2 and Synaptophysin (Syp). Quantification of Skp2 and Syp levels is demonstrated to the right. Scale bars are 25 μm. Comparison between groups was performed using Student's *t*-test. Bars indicate SEM. * $P < 0.05$, ** $P < 0.01$, *** $P < 0.001$, **** $P < 0.0001$. cBioPortal reference: Ren, Shancheng, et al. "Whole-genome and transcriptome sequencing of prostate cancer identify new genetic alterations driving disease progression." *European urology* 73.3 (2018): 322-339.

Supplementary Figure S4. SKP2 KD in C4-2B and 22Rv1 PCa cells. Quantification of qRT-PCR analysis relative to housekeeping gene beta-actin displaying mRNA levels for *SKP2* in C4-

2B and 22Rv1 cells upon *SKP2* knockdown (n=3 replicates). Comparison between groups was performed using Student's *t*-test. Bars indicate SEM. * P< 0.05, ** P< 0.01, *** P< 0.001, **** P<0.0001.

Supplementary Figure S5. SKP2 promotes K6- and K29-linked ubiquitination. (a-b) *In vivo* ubiquitination assay displayed an increase in ubiquitination for FOXA1 with wild-type (WT) Myc-SKP2, Flag tagged FOXA1 and HA-Ub in HEK293T cells. A decrease in FOXA1 ubiquitination was observed with lysine specific HA-ub K48 and K63. An increase in K6 and K29-linked ubiquitination of FOXA1 upon addition of Myc-SKP2 in HEK293T cells. Comparison between groups was performed using Student's *t*-test. Bars indicate SEM. * P< 0.05, ** P< 0.01, *** P< 0.001, **** P<0.0001.

Supplementary Figure S6. SKP2 overexpression increases FOXA1 ubiquitination. HEK293T cells were co-transfected with Myc-tagged SKP2, Flag-FOXA1 and HA-tagged ubiquitin. FOXA1 protein levels were quantitively defined. All samples were leveled to the same plasmid DNA concentration using an empty vector (EV) control and *In* vivo ubiquitination was performed. Comparison between groups was performed using Student's *t*-test. Bars indicate SEM. * P< 0.05, ** P< 0.01, *** P< 0.001, **** P<0.0001.

Supplementary Figure S7. SKP2 overexpression decreases FOXA1 protein by increasing FOXA1 ubiquitination. (a) Immunofluorescence (IF) images show the effects of SKP2 overexpression on FOXA1 in for C4-2B and 22Rv1 PCa cells. Cells were transfected with Myc-SKP2. After 24-hours, cells were fixed and immunostained with anti-Myc-SKP2 (green) and anti-FOXA1 (red) antibodies and counterstained with DAPI to visualize nuclei. White arrows represent high Myc-SKP2 and low FOXA1 protein levels. Scale bars are 25 μm. **(b-c)** *In vivo* ubiquitination assay for C4-2B and 22Rv1 PCa cells after Myc-SKP2 overexpression. Quantification represents the whole cell lysate (WCL) protein levels relative to housekeeping gene. Comparison between groups was performed using Student's *t*-test. Bars indicate SEM. * P< 0.05, ** P< 0.01, *** P< 0.001, **** P<0.0001.

Supplementary Figure S8. SKP2 KD decreases FOXA1 ubiquitination by increasing FOXA1 protein stability. (a-b) FOXA1 ubiquitination for C4-2B and 22Rv1 SKP2 knockdown (KD) cells and quantification of relative protein levels. **(c-d)** Cycloheximide chase (CHX;100

μg/mL) experiment at the indicated time points for C4-2B and 22Rv1 SKP2 KD cells. Comparison between groups was performed using Student's *t*-test. Bars indicate SEM. * P< 0.05, ** P< 0.01, *** P< 0.001, **** P<0.0001.

Supplementary Figure S9. Prostate tumors of *Pten*$^{pc-/-}$*; Trp53*$^{pc-/-}$*; Skp2*$^{-/-}$ mice have increased Foxa1 protein levels. (a) Immunofluorescence (IF) staining for luminal (CD24+) and basal (CD49f+) lineage markers in 1- and 4-week prostate organoids. Scale bars are 25 μm. **(b)** H&E and immunohistochemistry (IHC) staining for Skp2 and Foxa1 from anterior prostate (AP) mouse tissue from the indicated genotypes of mice at 3 months of age. Scale bars are 100 μm. **(c-d)** Quantification of Skp2 and Foxa1 positive cells in anterior prostate (AP). **(e)** Skp2 to Foxa1 ratio (Skp2:Foxa1) for IF staining. Comparison between groups was performed using Student's *t*-test. Bars indicate SEM. * P< 0.05, ** P< 0.01, *** P< 0.001, **** P<0.0001.

Supplementary Figure S10. SKP2 inhibition decreases FOXA1 ubiquitination. 22Rv1 FOXA1 ubiquitination proceeding inhibition with SZL P1-41 (0-10μm) for 24 hours and quantification of relative protein levels. Comparison between groups was performed using Student's *t*-test. Bars indicate SEM. * P< 0.05, ** P< 0.01, *** P< 0.001, **** P<0.0001.

Supplementary Figure S11. Ubiquitination of FOXA1 decreases after SKP2 KD and inhibition. (a) C4-2B mean fluorescence intensity (MFI) for ubiquitinated FOXA1. **(b)** 22Rv1 Percent and MFI for FOXA1 ubiquitination in upon SKP2 knockdown (KD) or treatment with SZL P1-41 (0-10μm) for 24 hours. Comparison between groups was performed using Student's *t*-test. Bars indicate SEM. * P< 0.05, ** P< 0.01, *** P< 0.001, **** P<0.0001.

Supplementary Figure S12. Colocalization of SKP2, FOXA1 and PCNA decreases after SKP2 inhibition in 22Rv1 xenograft mice. (a) Triple immunofluorescence (IF) staining showing colocalization of SKP2 (green), FOXA1 (red) and PCNA (purple) in 22Rv1 xenograft tissue either in the Vehicle or SZL P1-41. Scale bars are 25 μm. **(b-c)** 22Rv1 and C4-2B SKP2 to FOXA1 ratio (SKP2:FOXA1) for IF staining. **(d)** 22Rv1 xenograft immunohistochemistry (IHC) staining for FOXA1 and PCNA proceeding 30 days treatment with DMSO (Vehicle) or SZL P1-41 (SKP2 inhibitor; 30mg/kg; three times a week; intraperitoneal, i.p.). Scale bars are 100 μm. **(e)** IF images for luminal (CD24+) and basal (CD49f+) lineage markers in 22Rv1 vehicle and SZL P1-41 treated xenograft mice. Scale bars are 25 μm. Comparison between groups was

performed using Student’s *t*-test. Bars indicate SEM. * P< 0.05, ** P< 0.01, *** P< 0.001, **** P<0.0001.

Supplementary Figure S13. FOXA1 ubiquitination by SKP2 occurs in the C-terminal TAD. (a) A schematic displays a series of FOXA1 subdomains. TAD, transactivating domain; FKHD, forkhead domain, where FOXA1 undergoes DNA binding; C-terminal TAD, where protein interaction can occur. **(b)** *In vivo* ubiquitination of FOXA1 and subdomains in HEK293T cells transfected with HA-Ub, Myc-tagged SKP2 and His-FOXA1 wild-type, WT (1-466 amino acids), along with various FOXA1 domain constructs. (c) SKP2 pull-down displays the SKP2-FOXA1 domain interactions.

Supplementary Figure S14. Ubpred predicted ubiquitination sites for FOXA1. Predicted ubiquitination sites for FOXA1 from UbPred (http://www.ubpred.org/). The FOXA1 primary amino acid sequence was used. Conserved lysine ubiquitination sites on FOXA1 and the amino acid sequence similarity for Rat and Human FOXA1 according to NCBI Blast and ClustalW. Percent similarity between human and rat FOXA1 amino acid sequence according to NCBI Blast and ClustalW.

Supplementary Figure S15. The effects of FOXA1 mutation on ubiquitination. (a) A decrease in the ubiquitination of FOXA1 is observed when lysine 414 and 418 are individually mutated. **(b)** Representative histogram depicts the profile of FOXA1 ubiquitination in HEK293T cells. The percentages and mean fluorescence intensity (MFIs) of FOXA1 ubiquitination in HEK293T cells upon overexpression of Myc-SKP2, HA-Ub and His-FOXA1 wild-type (WT) or mutants. Comparison between groups was performed using Student’s *t*-test. Bars indicate SEM. * P< 0.05, ** P< 0.01, *** P< 0.001, **** P<0.0001.

Supplementary Figure S16. FOXA1 protein levels increase upon lysosomal inhibition. 22Rv1 cells were exposed to MG132 and Chloroquine treatment with the indicated concentrations (μm). Lysates were analyzed for FOXA1 protein levels. Comparison between groups was performed using Student’s *t*-test. Bars indicate SEM. * P< 0.05, ** P< 0.01, *** P< 0.001, **** P<0.0001.

Supplementary Figure S17. Lysosomal inhibition abrogates effects of SKP2 overexpression on FOXA1. HEK293T cells were co-transfected with wild-type (WT) Myc-SKP2, Flag tagged FOXA1 and HA-Ub and subjected to MG132 and Chloroquine treatment. Lysates were analyzed for FOXA1 protein levels. Comparison between groups was performed using Student's *t*-test. Bars indicate SEM. * P< 0.05, ** P< 0.01, *** P< 0.001, **** P<0.0001.

Supplementary Figure S18. SKP2, FOXA1 and LAMP2 colocalize in 22Rv1 cells. 22Rv1 cells were transfected with wild-type (WT) Myc-SKP2 and immunofluorescence (IF) was performed to visualize increases in LAMP2 (lysosome-associated membrane glycoprotein) levels. White arrows represent LAMP2, SKP2 and FOXA1 protein colocalization. Scale bars are 20 μm.

Supplementary Figure S19. *Skp2* mRNA levels decrease in *Pten/Trp53/Skp2* triple null MEF's. qRT-PCR results displaying relative mRNA levels for Skp2 (housekeeping gene is beta-actin) in the indicated genotypes of Mouse embryonic fibroblasts (MEF's). Comparison between groups was performed using Student's *t*-test. Bars indicate SEM. * P< 0.05, ** P< 0.01, *** P< 0.001, **** P<0.0001.

Supplementary Figure S20. Protein stability for Foxa1 increases in *Pten/Trp53/Skp2* triple null MEF's. Pten$^{\triangle/\triangle}$; Trp53$^{\triangle/\triangle}$ and Pten$^{\triangle/\triangle}$; Trp53$^{\triangle/\triangle}$; Skp2$^{-/-}$ mouse embryonic fibroblasts (MEF's) treated with Cycloheximide (CHX; 100μg/mL) protein synthesis inhibitor for the indicated time points (h, hours). Following the samples were collected for western blot analysis. FOXA1 protein intensity is displayed in the corresponding plot. Comparison between groups was performed using Student's *t*-test. Bars indicate SEM. * P< 0.05, ** P< 0.01, *** P< 0.001, **** P<0.0001.

Supplementary Table S1: Genotyping PCR primer sequences

Supplementary Table S2: Real-time quantitative PCR and shRNA primer sequences
